# Comparative Numerical Assessment of Fabrication-Aware Modelling Simplifications in PCB, Screen-Printed and Embroidered Split-Ring FSS Structures

**DOI:** 10.3390/s26216687

**Published:** 2026-10-22

**Authors:** Alan Michalczyk, Tomasz Rybicki, Iwona Karbownik, Wiktor Śmiechowski, Marcin Barburski, Tsegaye Lemmi, Hasan Riaz Tahir

**Affiliations:** 1 Institute of Architecture of Textiles, Faculty of Material Technologies and Textile Design, Lodz University of Technology, 116 Zeromskiego St., 90-543 Lodz, Poland; 2 Institute of Automatic Control, Faculty of Electrical, Electronic, Computer and Control Engineering, Lodz University of Technology, 18 Stefanowskiego St., 90-537 Lodz, Poland

**Keywords:** frequency selective surfaces (FSS), fabrication-aware modelling, split-ring resonator, textile electronics, screen-printing, embroidered shielding materials, PCB technology

## Abstract

This paper investigates how geometric and material modelling fidelity affects the simulated electromagnetic performance of a split-ring frequency selective surface (FSS) fabricated using three technologies: rigid printed circuit boards (PCBs), screen-printing, and embroidery. Within each technology, progressively simplified models are compared to identify which fabrication-related features can be omitted without substantially altering the simulated resonant response. For PCB, three model variants are considered: a full layered structure, a model without solder mask and copper fill, and a bare-copper model without dielectric layers. For screen-printing, the conductive paste and textile substrate are represented using uneven-surface and solid approximations. For embroidery, detailed-thread geometry is compared with a simplified solid representation. The effects of these modelling choices are evaluated using bandwidth, fractional bandwidth, resonance frequency, and transmission coefficient (S21) resonance depth for the fundamental transverse electric (TE) and transverse magnetic (TM) responses. The results demonstrate that modelling sensitivity is strongly technology dependent. For PCBs, the dielectric representation has the strongest influence on the overall resonance pattern. Omitting the solder mask and copper fill has a limited effect on the TE resonance frequency, but produces more noticeable changes in the TM response, demonstrating that the impact of secondary layers depends on the polarisation and the performance metric considered. Screen-printing shows moderate sensitivity, particularly in resonance depth. The largest discrepancies occur for embroidery, where thread-level simplification produces pronounced TE/TM-dependent resonance shifts and additional resonances absent from the solid model. Based on these results, comparative observations are presented regarding the suitability of different modelling representations during numerical design studies.

## 1. Introduction

Frequency selective surfaces (FSS) are two-dimensional periodic electromagnetic structures that exhibit frequency dependent transmission and reflection characteristics, which can be used as filters of electromagnetic waves [1,2,3,4]. By appropriately selecting the geometry of the unit cell, substrate properties, and periodicity, FSS can be designed to operate as band pass, band stop, or multiband structures [1,2,3,4,5], enabling applications ranging from radomes [6] and electromagnetic shielding [1,7] to wireless communication systems [1,3,8,9], sensing technologies [1,3], and other advanced electromagnetic wave-control applications [10]. Among the various FSS geometries, split-ring resonator unit cells [10,11,12,13] have been widely investigated due to their well-defined resonant behaviour and sensitivity to geometrical parameters. Therefore, the split-ring unit cell represents a suitable reference structure for evaluating the influence of fabrication-dependent modelling simplifications, as its electromagnetic response is highly sensitive to variations in both geometric and material parameters.

With the continued deployment of 5G wireless networks [8,9] and the increasing demand for electromagnetic compatibility [1,7], lightweight filtering structures operating over precisely controlled frequency bands have become increasingly important [1,3]. The electromagnetic behaviour of an FSS is determined not only by the geometry of its conductive elements [1,2,4] but also by the surrounding dielectric environment [2,6,14] and fabrication technology [15,16,17,18,19,20,21,22,23]. Previous studies have demonstrated that electromagnetic performance is strongly influenced by substrate properties, multilayer configurations, and geometrical parameters [2,14,24,25,26,27]. Consequently, electromagnetic simulations have become an essential stage of the design process [1,2,3,4], allowing optimisation before prototype fabrication to significantly reduce development time and cost [2,4].

Although numerical modelling techniques for FSS have been extensively investigated [1,2,3,4,25,28], most published work assumes that the simulated geometry accurately represents the manufactured structure. In practice, however, the achievable level of geometric fidelity depends strongly on the fabrication technology [15,16,17,18,19,20,21,22,23,29]. Conventional printed circuit boards (PCB) can generally be modelled with well-defined dielectric layers and conductor geometries [2,6,14], whereas textile-based implementations, including screen-printing and embroidery, require various geometric approximations because of material heterogeneity and structure complexity [15,16,17,21,22,23]. Simplified representations are therefore frequently adopted to reduce computational cost [21,23] or to satisfy the limitations of commercial electromagnetic solvers [21,23]. Despite their widespread use, the influence of these simplifications on the predicted electromagnetic response has received relatively little systematic attention [17,18,19,21,23].

Several studies have investigated optimisation of unit-cell geometries [5,25,26], equivalent circuit models [25,28], bandwidth enhancement [14,24], and angular stability [30,31,32,33]. However, comparatively few publications examine how fabrication-aware geometric simplifications themselves influence simulation accuracy or whether the same modelling assumptions remain valid across fundamentally different manufacturing technologies [17,18,19,20,21,22,23]. This issue is particularly relevant for textile electronics, where conductive threads, printed conductive inks, and flexible dielectric substrates exhibit substantially different physical characteristics from conventional PCB laminates [15,16,17,21,22,23].

To address this gap, this work investigates how the level of geometric and material fidelity influences the simulated performance of a split-ring FSS fabricated using three representative technologies: PCBs, screen-printing on textile substrates, and embroidery with conductive threads. Instead of comparing unrelated fabrication methods, each technology is analysed through progressively simplified models. The resulting changes in resonance frequency, transmission and reflection characteristics, and modal behaviour are evaluated separately for both TE and TM polarisations. Based on these comparisons, the present work investigates the relative influence of commonly adopted fabrication-aware modelling simplifications on the simulated electromagnetic response of FSS structures and provides comparative observations regarding their suitability for different stages of numerical design studies. Although the present study is entirely simulation-based, it provides a framework for future experimental validation of fabrication-aware modelling strategies for both rigid and flexible FSS implementations.

## 2. Methods

### 2.1. Unit-Cell Geometry

To allow a fair comparison of modelling fidelity, a single split-ring unit-cell geometry is used as the common reference across all three fabrication routes investigated in this study: PCB, screen-printing, and embroidery. Using a shared geometry ensures that any differences observed between technologies, or between levels of model detail within a technology, can be either attributed to the difference of fabrication method or modelling choices under investigation rather than to differences in given dimensions in resonator design.

**Figure 1:**
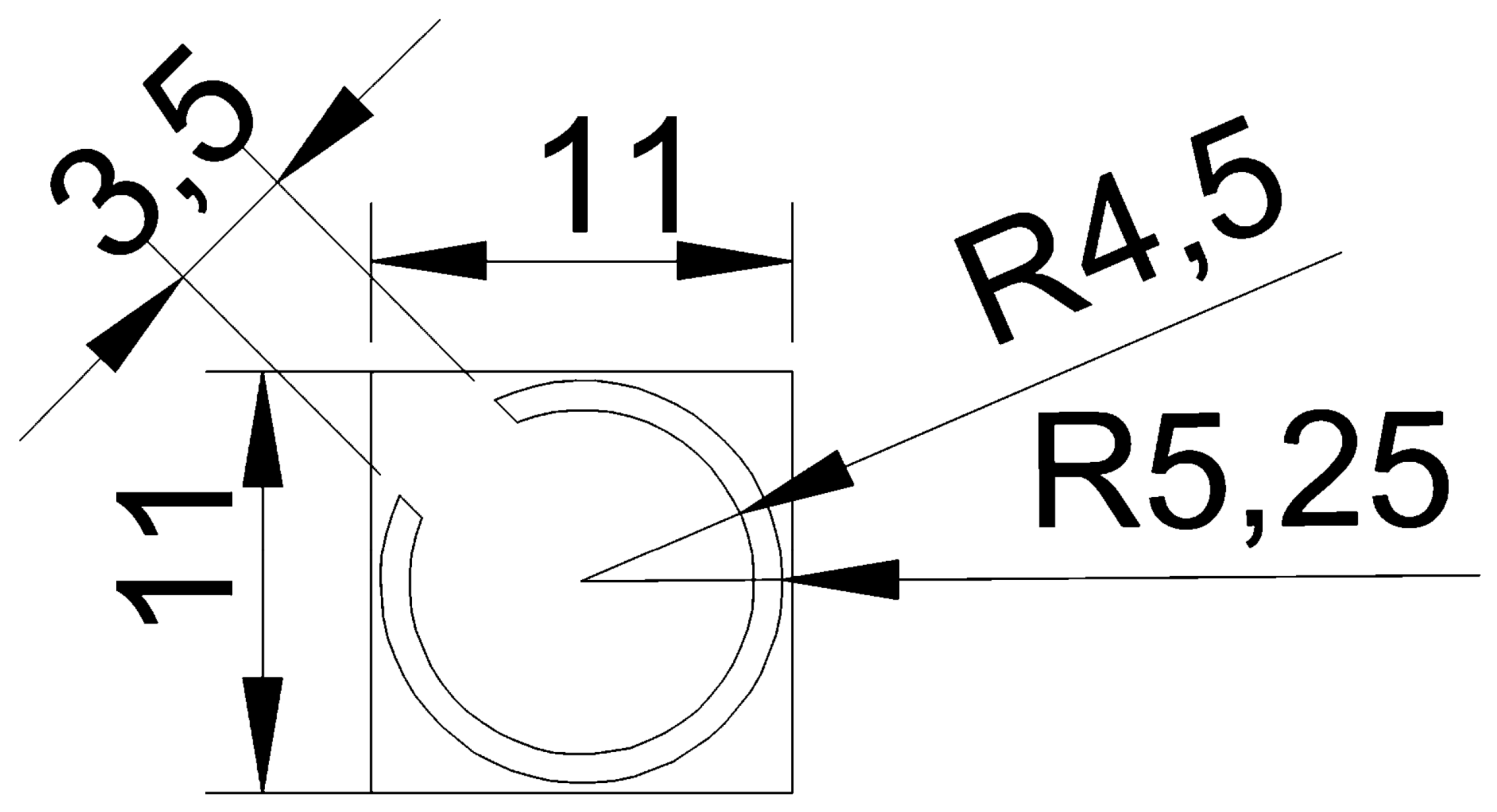
Dimensions (in millimetres) of a single geometry cell.

The unit cell presented in the Figure 1 consisted of a split-ring conductor and dimensions of the entire unit cell are: 11 mm × 11 mm × 1.6 mm, split-ring inner radius of 4.5 mm, split-ring outer radius of 5.25 mm and split-ring gap width of 3.5 mm. All dimensions were within ±2% of their nominal values. This dimensional tolerance might introduce a corresponding variation in the simulated electromagnetic response, particularly in the resonance frequency, bandwidth, and resonance depth. Therefore, the reported ±2% dimensional accuracy should be considered as a potential source of variation between the nominal simulation model and the physically fabricated structure rather than as a direct ±2% uncertainty in the electromagnetic response.

Conductor was realised, depending on the fabrication route, as copper trace (PCB), conductive paste (screen-printing), or conductive thread (embroidery), each deposited on or integrated into a substrate whose material properties were specific to the corresponding technology and are detailed later.

The split-ring topology was selected for its compact electrical size relative to the unit cell period, its well-characterised dual-resonance behaviour, and its established suitability for realisation on non-rigid and textile substrates using embroidery and printing processes. Two resonant modes were supported by the structure within the frequency range of interest, referred to throughout this paper as Mode TE and Mode TM, and were treated as separate points of comparison in all subsequent analysis, since there was no certainty that fabrication-induced deviations affect both modes equally.

The unit-cell geometry was, in turn, constrained by the resolution limits of the embroidery process [29] rather than by electromagnetic optimisation alone. Modelling the ring as an equivalent series LC circuit [12] could be expressed with the equation:

(1) $$\begin{array}{c} f_{0}=\frac{1}{2\pi \sqrt{LC}} \end{array}$$

where:

- $f_{0}$—resonant frequency,
- L—equivalent inductance of the ring,
- C—equivalent capacitance of the ring.

For non-magnetic materials, the inductance of the ring could be approximated as a function of the ring radius and conductor radius:

(2) $$\begin{array}{c} L\;\approx \;\mu_{0}R\;\left(ln\left(\frac{8R}{r}\right)-\frac{7}{4}\right) \end{array}$$

where $\mu_{0\;}$ is the permeability of free space ($4\pi \times 10^{-7}$ H/m), R is the ring radius, and r is the conductor (wire or thread) radius.

The capacitance was given by:

(3) $$\begin{array}{c} C\;\approx \;\pi^{2}\varepsilon_{0}R\;{\left[3\;ln\left(\frac{R}{r}\right)\right]}^{-1}+\;\left(\frac{\pi \varepsilon_{0}r^{2}}{d}\right)\;\left[1\;+\;\left(\frac{d}{\pi r}\right)\left(ln\left(\frac{16\pi r}{d}\right)\;-\;1\right)\right] \end{array}$$

where $\varepsilon_{0}$ is the permittivity of free space, R is the ring radius, r is the conductor (wire or thread) radius, and d is the substrate thickness.

With $\varepsilon_{0}$ and $\mu_{0}$ fixed, Equations (2) and (3) show that the resonant frequency $f_{0}$ is governed by three geometric parameters: R, r, and d.

For embroidery, r and d were fixed at the smallest values that could be produced reliably and repeatably, considering the minimum thread diameter, stitch resolution, and substrate compliance. The ring radius R was then determined iteratively to obtain the target resonant frequency for each mode. This geometry was subsequently used as the common reference for evaluating fabrication-induced deviations in Mode TE and Mode TM.

### 2.2. Simulation Setup

Numerical simulations were performed using ANSYS Electronics Desktop 2025 R1 (Ansys, Inc., Canonsburg, PA, USA) (HFSS) on a workstation running Microsoft Windows 11 Pro (64-bit) (Microsoft Corporation, Redmond, WA, USA). The workstation was equipped with an AMD (Advanced Micro Devices, Inc., Santa Clara, CA, USA) Ryzen 9 9950X3D 16-Core Processor (16 physical cores, 32 logical processors, base clock 4.3 GHz) and 96 GB of RAM. Electromagnetic simulations were executed using one HFSS solver licence with 10 HPC licences enabled for parallel computation, allowing efficient utilisation of the available CPU resources.

All electromagnetic simulations were performed using the finite element method (FEM), using an interpolating frequency sweep. The simulation domain was defined as a single unit cell bounded by master-slave periodic boundary conditions on the four lateral faces, enforcing phase continuity between adjacent cells and enabling representation of an infinite periodic array from a single-cell model. Excitation was provided by Floquet ports placed on the faces perpendicular to the direction of wave propagation, each supporting the fundamental TE and TM modes.

**Figure 2:**
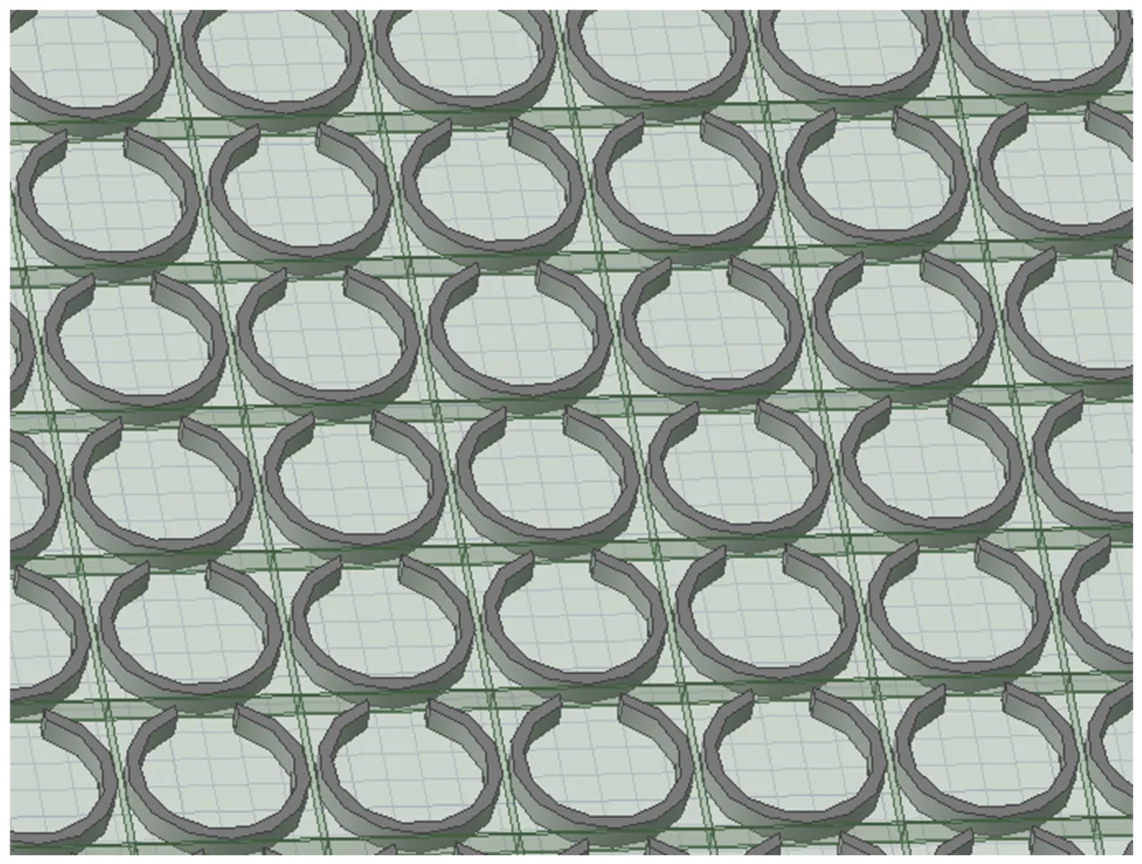
Illustration of the Floquet port working principle (Floquet port assumes an infinite periodic array of unit cells in the x- and y-axis directions).

The working principle of the Floquet ports is illustrated in Figure 2.

In practice, only one single unit cell was modelled, while the Floquet boundary conditions mathematically extend it into an infinitely periodic array. In HFSS, the number of Floquet modes included in the analysis was selected based on their propagation characteristics. Only the fundamental TE_00_ and TM_00_ modes exhibited zero attenuation. These modes are hereafter referred to without the subscripts and represent the propagating modes considered in this study. All higher-order Floquet modes showed a non-zero attenuation constant, confirming that they remained evanescent within the investigated frequency range and did not contribute to power transmission.

All simulations presented in this paper correspond to normal incidence. All simulations were performed using the same unit-cell orientation, with the gap consistently aligned along the y-axis. The frequency sweep range is set to 0–15 GHz, chosen to capture resonances with sufficient margin on either side of the response to characterise the full bandwidth. The primary simulation outputs were the S parameters, S11 (reflection coefficient) and S21 (transmission coefficient), for Mode TE and Mode TM in each simulated configuration. The convergence of the adaptive-meshing process was governed by the software’s automated settings. No manual local mesh treatments were applied to the structures. Due to the diverse geometric complexities of the fabrication technologies, adaptive-pass history, final mesh statistics, and the total number of matrix unknowns naturally scaled and varied across the models. Crucially, the mesh adaptation process exhibited smooth convergence with a clear absence of oscillations or instabilities between consecutive passes. No significant instabilities were observed during the adaptive-meshing process, and all models satisfied the convergence criteria imposed by the solver. A dedicated mesh-refinement study was outside the scope of the present work, and therefore the reported numerical results should be interpreted within the adopted adaptive-meshing framework.

The frequency sweep was performed using 251 frequency points over the 0–15 GHz range with the HFSS interpolating solver and the higher-speed automatic solver setting. The same periodic boundary conditions and Floquet-port configuration were applied to all model variants. The y-axis was aligned with the split-ring gap, the x-axis was oriented perpendicular to the gap within the FSS plane, and the z-axis was directed normal to the FSS plane. Floquet Port 1, located above the FSS, was excited with a power of 1 W and a phase of 0°, while the second Floquet port was located below the structure. These values describe the frequency sampling resolution and should not be interpreted as the intrinsic numerical error of the FEM solution, which depends on factors such as mesh convergence, adaptive solution criteria, and solver convergence.

### 2.3. Model Variants

One or more additional variants were constructed by progressively removing geometric or material detail. The specific variants considered for each technology were summarised in Table 1 and described in full below:

- PCB: (i) a full-layer stack comprising the dielectric substrate and solder mask; (ii) a structure without the solder mask and fill layers; and (iii) a copper trace without dielectric representation.
- Screen-printing: (i) a conductive paste layer with an uneven surface deposited on an uneven textile substrate; (ii) a conductive paste layer with an uneven surface deposited on a solid textile substrate; and (iii) a conductive paste layer with a solid surface deposited on a solid textile substrate.
- Embroidery: (i) a detailed representation of conductive threads interwoven with a solid, homogeneous textile substrate; and (ii) a solid, homogeneous conductive region formed by conductive threads embedded within a solid, homogeneous textile substrate.

The screen-printing technology was the only investigated approach in which the overall thickness was slightly more than 1.6 mm (printed electroconductive paste layer, equal to 0.03 mm, was added on top of the substrate).

The complete manufactured stack, referred to as the full-stack PCB model, consisted of a dielectric substrate with a thickness of 1.51 mm, a copper conductor layer with a thickness of 0.035 mm forming the split-ring pattern, and a solder mask layer with a thickness of 0.01 mm covering the exposed conductor and substrate, in accordance with standard PCB fabrication practices.

**Figure 3:**
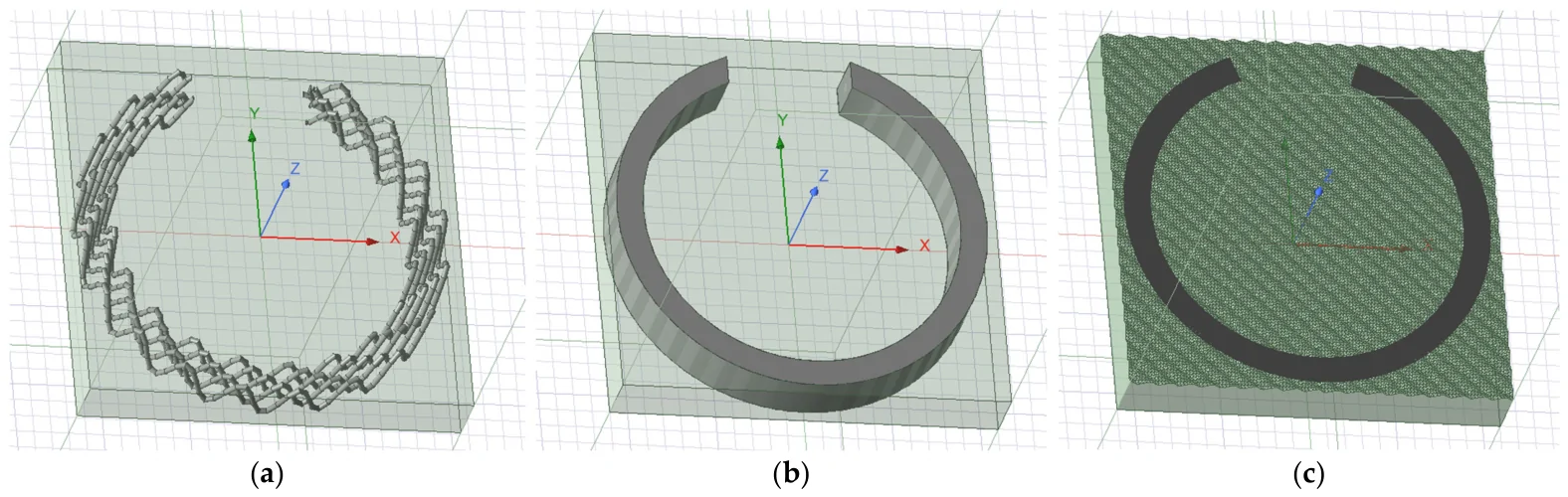
Visual view of embroidery models. (**a**) Detailed-thread model. (**b**) Solid model. (**c**) Screen-printing, uneven surfaces model.

To facilitate the visualisation of the embroidery models, all analysed configurations are presented in Figure 3.

Materials used for representation in HFSS models per technology were as follows:

- PCB models: copper, FR4 epoxy and core,
- screen-printing model: numerical equivalent of cotton, silver paste,
- embroidery models: numerical equivalent of cotton, silver treads.

The substrate for screen-printing and embroidery samples was cotton fabric with a twill weave structure [34,35]. Therefore, material parameters in the simulation software were adopted as presented in Table 2 according to the scientific articles, respectively for cotton [34,36], silver threads [37] and silver paste [38]. The adopted values represent literature-based approximations selected for comparative numerical evaluation and should not be interpreted as experimentally characterised properties of the fabricated structures.

The material parameters used for the textile implementations should be considered in the context of the fabrication-aware modelling objective of this study. For the embroidered implementation, the actual structure consisted of cotton twill fabric. The material representation adopted here should be regarded as an approximation suitable for assessing the influence of fabrication-related geometrical details, rather than as a fully experimentally validated representation of the fabricated textile assembly.

The PCB structure was the most controllable as all the layers, materials and its electromagnetic properties were imported from PCB board layout prepared in KiCad v9.0.4 (KiCad Project, open-source) software, directly into ANSYS and then into ANSYS HFSS. On the other hand, given the intrinsic difficulty of geometrically resolving thread cross-sections in a periodic solver model, embroidery was evaluated at the least accurate level of detail out of the three technologies presented.

**Figure 4:**
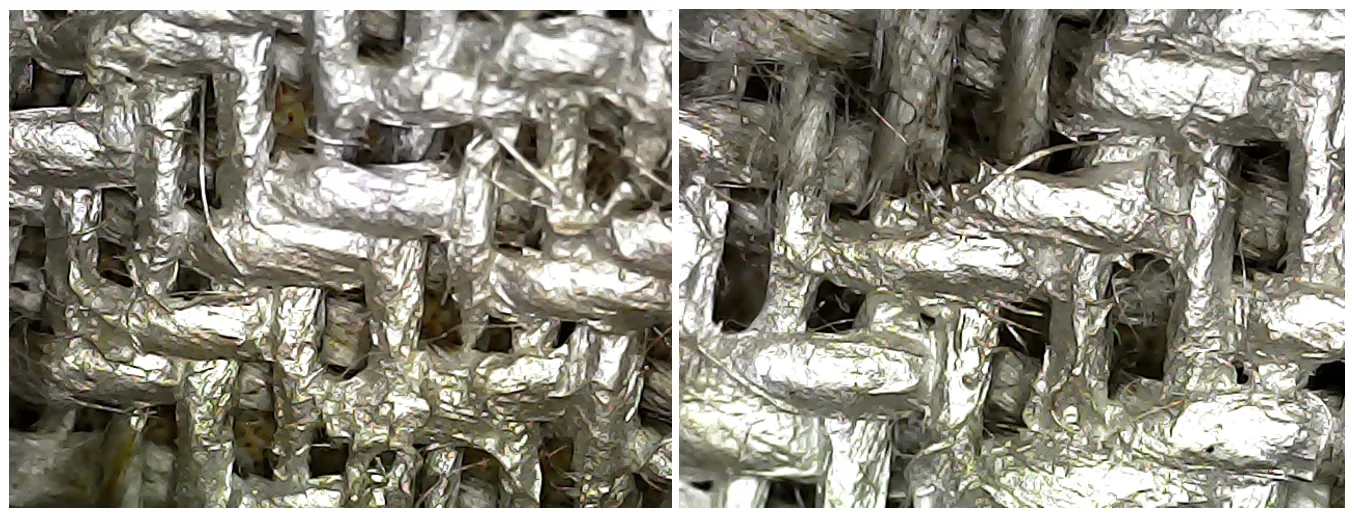
Exemplary microscopic images illustrating the surface morphology of fabric printed with a silver-containing paste.

The uneven paste-surface geometry was introduced to qualitatively reproduce the irregular surface characteristics resulting from the screen-printing process. Microscopic images illustrating the surface morphology is presented in Figure 4. As no additional surface measurements were performed, the model does not represent an exact reconstruction of the actual surface profile. Instead, it provides a simplified, yet representative approximation of the surface irregularities observed in the manually screen-printed paste.

**Figure 5:**
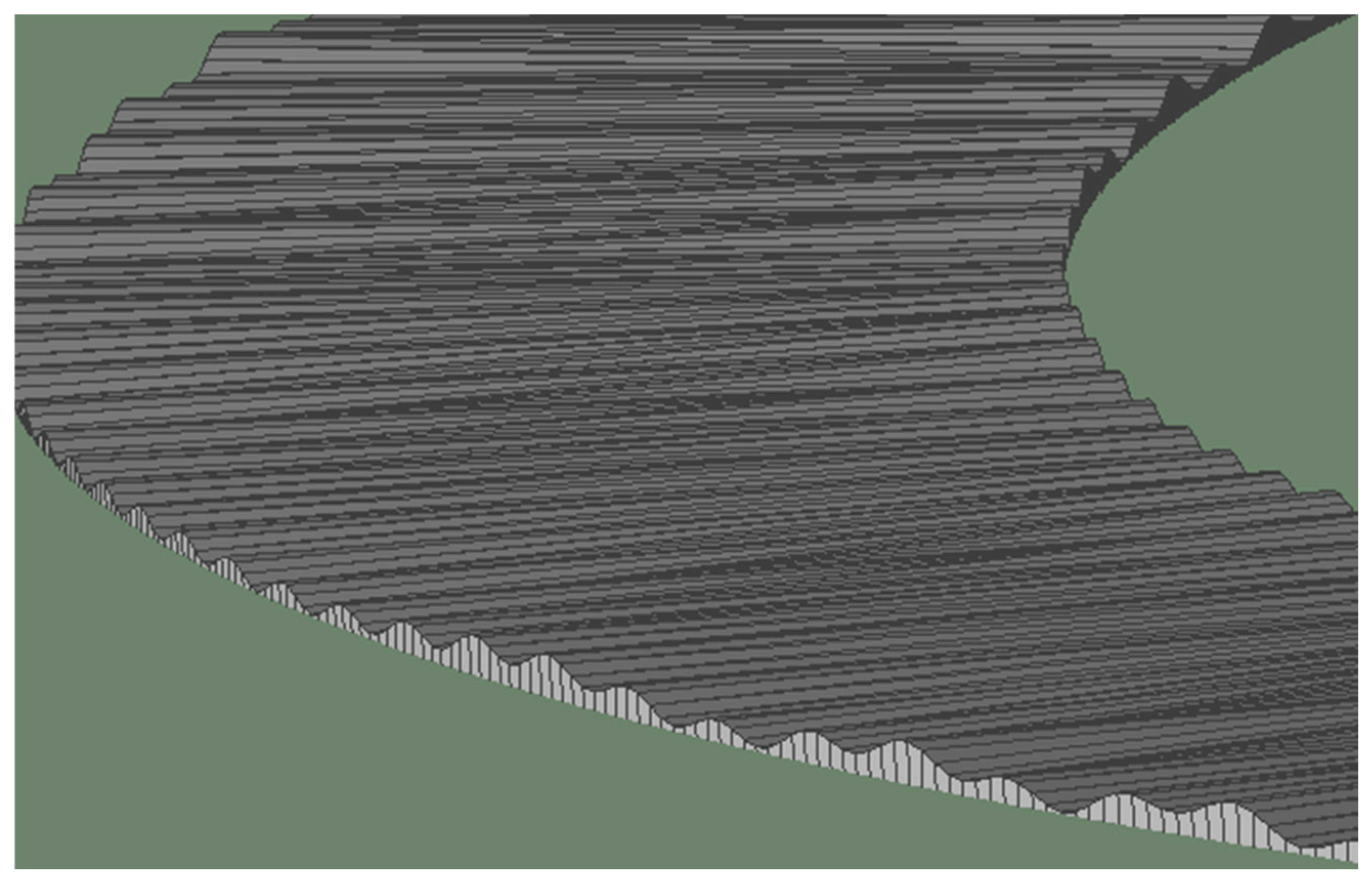
Visual representation of uneven paste-surface model.

In contrast to studies focusing on individual manufacturing defects [16,17,18], the present work introduces stochastic surface irregularities into the numerical model to represent the non-uniform geometry of the screen-printed conductive layer deposited on the textile substrate. The introduced irregularities are not intended to reproduce a specific defect observed in a fabricated sample, but rather to provide a representative approximation of the surface variations associated with the printing process. For clarity, a detailed view of the corresponding uneven-surface model is presented in Figure 5.

For the embroidered implementation, the model was based on the actual conductive yarns considered for fabrication, namely Shieldex^®^ (Statex Produktions und Vertriebs GmbH, Bremen, Germany) Monofil 150 µm, Shieldex^®^ Monofil 80 µm, and Shieldex^®^ 117/17 dtex. The detailed model incorporated the nominal yarn diameters, stitch spacing, number of stitches, yarn consumption, and the resulting yarn fill factor, thereby accounting for the principal geometrical characteristics of the conductive textile structure. These fabrication parameters were consistent with the embroidery implementation presented in related work by the authors [39]. The conductive yarns were not treated as homogeneous bulk-silver wires; rather, their geometry was explicitly represented, while the conductive material was approximated using the available electromagnetic material model.

For the screen-printed implementation, experimentally characterised electromagnetic properties of the specific silver paste and printed layer were not available. The use of silver threads and silver paste properties were therefore adopted according to the scientific literature [34,36,37,38] and described in Table 2. This distinction is important because the electrical properties of screen-printed conductors depend strongly on the paste formulation, substrate morphology, printed-layer thickness, printing conditions, and curing process. The adopted material models should therefore be interpreted as controlled reference models used to isolate the influence of fabrication-related geometrical simplifications. The resulting numerical values describe the investigated modelling assumptions and should not be interpreted as experimentally calibrated predictions of a specific commercial paste, textile substrate, or embroidered structure. Experimental characterisation of substrate parameters remains necessary for quantitative validation and was identified as an important direction for future work.

**Figure 6:**
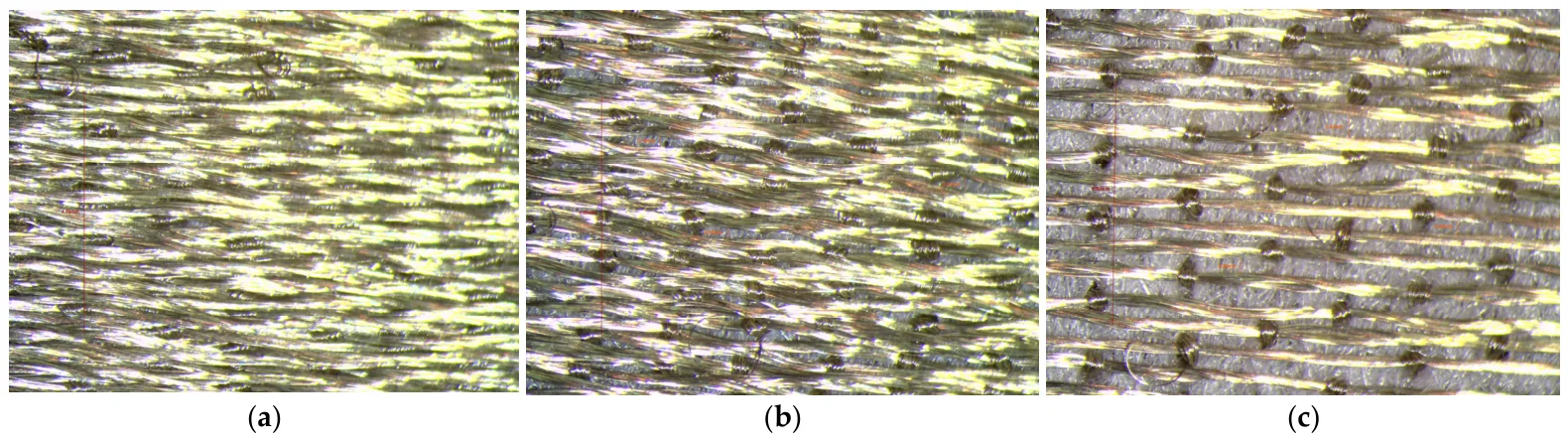
Visual view of embroidery sample with Multifilament 117/17 dtex yarn. (**a**) 2-unit gap. (**b**) 4-unit gap. (**c**) 8-unit gap.

**Figure 7:**
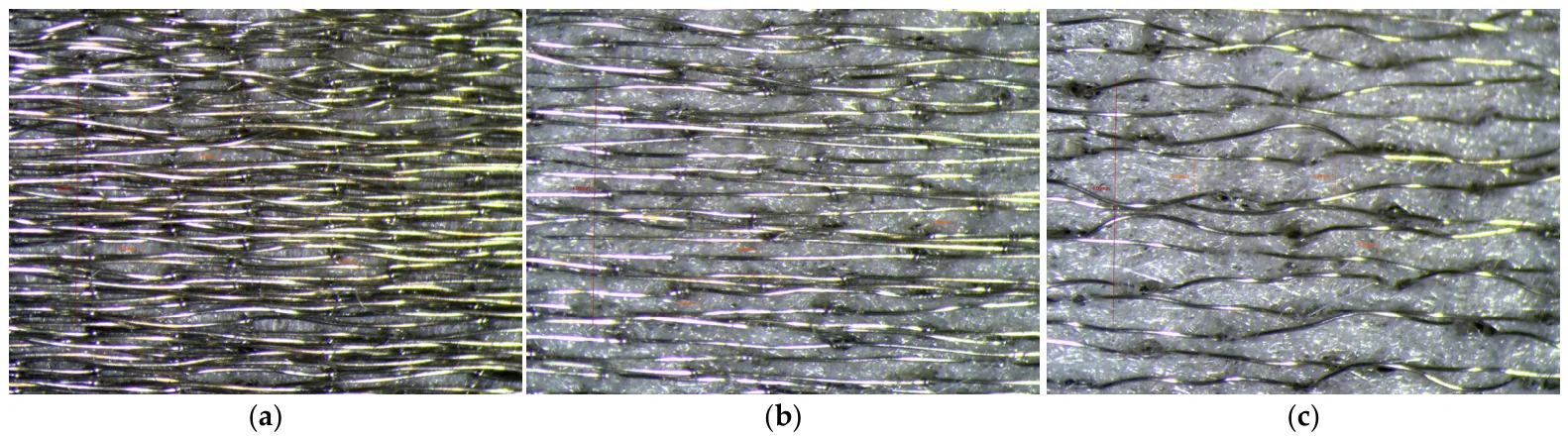
Visual view of embroidery sample with Monofilament 80 µ yarn. (**a**) 2-unit gap. (**b**) 4-unit gap. (**c**) 8-unit gap.

The embroidered FSS was modelled using the geometrical parameters of the intended fabrication process rather than as an arbitrarily defined conductive pattern. Three conductive yarns were considered: Shieldex^®^ Monofil 150 µm, Shieldex^®^ Monofil 80 µm, and Shieldex^®^ Multifil 117/17 dtex, presented in Figure 6 and Figure 7 for different density of threads. The embroidery geometry was defined by the stitch spacing, number of stitches, yarn consumption, and yarn cross-sectional dimensions. For stitch spacings of 2, 4, and 8 units, the corresponding numbers of stitches were 300, 148, and 80, with total yarn consumptions of 3.00, 1.47, and 0.80 m, respectively. The calculated projected yarn fill factors were dependent on the yarn type and stitch spacing; for example, for the 117/17 dtex yarn they were 30.6%, 15.1%, and 8.16% for the three investigated spacings, respectively, while for the 150 µm monofilament they were 13.5%, 6.66%, and 3.60%. The theoretical cross-sectional distribution of the embroidered yarn was approximately 80% on the upper side and 20% on the lower side. The embroidery was applied to a cotton fabric with a twill weave. These parameters establish the geometrical and material basis of the detailed-thread model and ensure that the conductive area and yarn distribution are determined by the intended fabrication geometry rather than by an arbitrary electromagnetic approximation. The simplified solid model, in contrast, represents a continuous conductive region occupying the nominal FSS pattern, and therefore does not preserve the local voids, yarn crossings, inter-thread spacing, or three-dimensional current paths of the detailed embroidery. It is consequently treated as a geometrical reference model rather than as an electrically equivalent homogenised representation. The comparison between the two models therefore evaluates the electromagnetic consequence of replacing the fabrication-resolved conductive geometry with a continuous approximation, while acknowledging that the two representations do not possess identical conductive filling or local current topology.

### 2.4. Comparison Metrics

To quantify the effect of model simplification in a consistent way across all technologies and both resonant modes, four comparison metrics were defined, each computed relative to the reference (highest-detail numerical representation considered in this study) variant available for a given technology:

- Resonance frequency: The difference between the resonance frequency of the simplified model and that of the reference model, for each mode and each of S11/S21, expressed in MHz,
- Resonance amplitude: The difference in the magnitude of the S-parameter at resonance frequency (attenuation depth for S21, reflection minimum/maximum for S11) between the simplified model and the reference model, expressed in dB,
- Bandwidth: Frequency range between the lower and upper cutoff frequencies of the resonant feature, within which the specified performance criterion was satisfied,
- Fractional bandwidth: Used to describe the relative bandwidth of the resonant feature and to enable a fair comparison between different operating frequencies. The bandwidth threshold used for defining the operating bandwidth is kept constant across all variants of the same technology to ensure consistent comparability. Bandwidth was evaluated using a −10 dB transmission threshold, which ensured that the operating band could be consistently identified across all investigated structures and modelling variants.

The fractional bandwidth (FBW) was expressed as the ratio of the absolute bandwidth to the corresponding centre frequency and is calculated according to:

(4) $$\begin{array}{c} FBW=\frac{\left(f_{H}-f_{L}\right)}{fc}\times 100\%\; \end{array}$$

where f_H_ and f_L_ represented the upper and lower frequency limits of the operating bandwidth, respectively, while f_c_ is the centre frequency of the considered frequency range. These four metrics were reported together for every model comparison, since a simplification may leave the resonance frequency largely unaffected while still distorting the depth or width of the response, or the other way around. Considering all four jointly was necessary to determine whether a given simplification was acceptable for design purposes. A simplification may be regarded as producing limited deviation if all these metrics remain within a predefined threshold.

To ensure a consistent comparison between the investigated model variants, the primary TE and TM resonances were identified as the dominant transmission minima associated with the resonance within the corresponding frequency range. Only resonances directly associated with these primary TE and TM responses were considered when determining the resonance frequency, resonance depth, bandwidth, and fractional bandwidth. Additional local resonances occurring outside the primary response bands were treated as secondary resonances and were not included in the bandwidth comparison.

## 3. Results of Parametric Simulation

### 3.1. PCB Board

The PCB case was treated in this study as the case in which the effect of removing model detail could be examined with the least ambiguity about whether any observed deviation originates from the simplification itself or from fabrication uncertainty. Three progressively simplified model variants were considered, from the most complete representation of the fabricated board to the least. Full-stack variant was treated as the reference against which the three subsequent simplifications were measured, since it most closely reflects the manufactured board.

**Figure 8:**
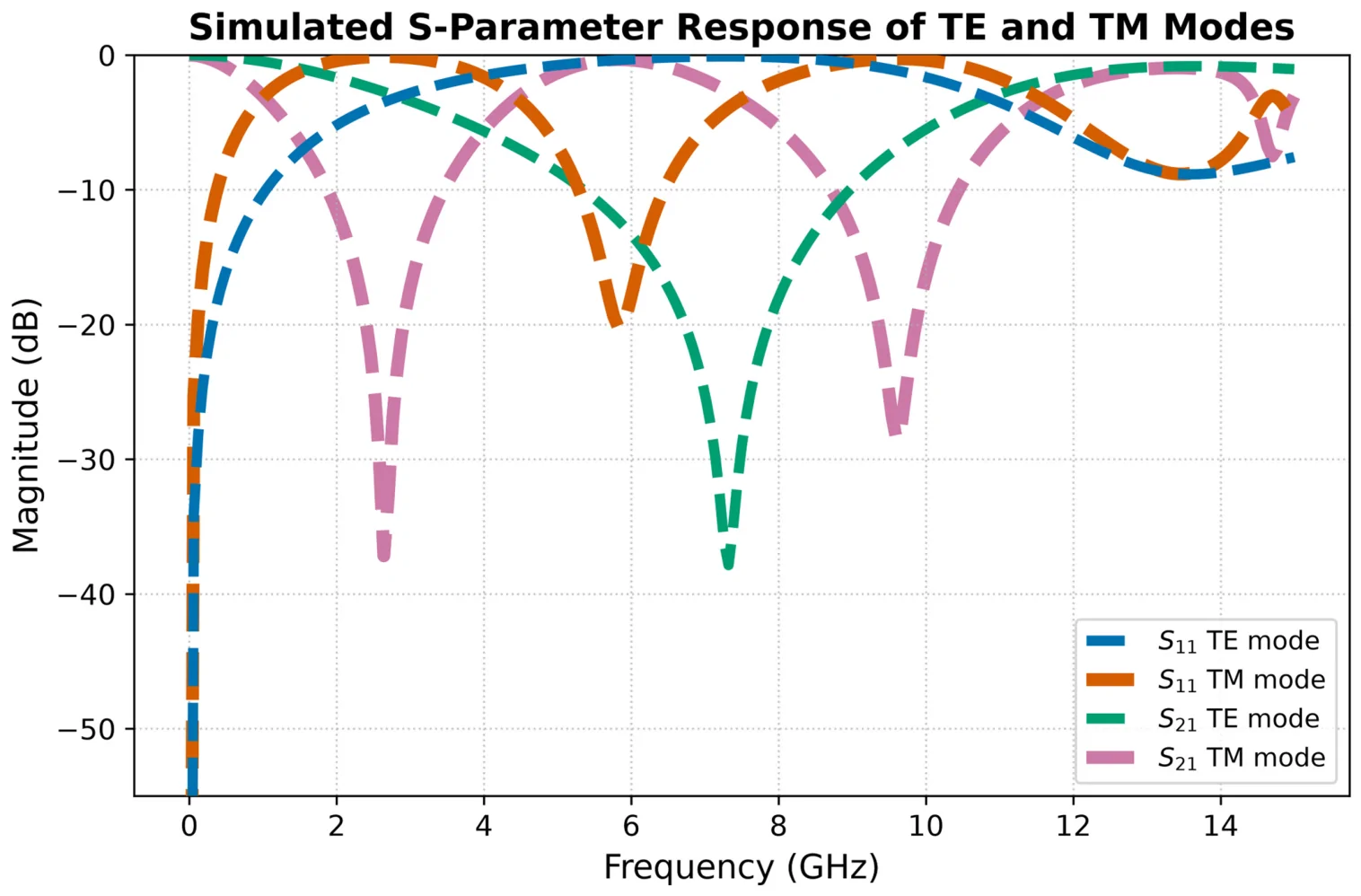
Simulated S11 and S21 response of the full-stack PCB model for TE and TM modes.

For the full-stack PCB model (Figure 8), resonance frequencies of 7.32 GHz (TE mode) and 2.64 GHz (TM mode) were observed in the S21 response, with corresponding attenuation depths of −37.86 dB and −37.17 dB, respectively. At the −10 dB level, the TE and TM modes exhibited bandwidths of 3.62 GHz and 1.58 GHz, respectively. These bandwidths corresponded to frequency ranges of 5.35–8.97 GHz for the TE mode and 1.88–3.46 GHz for the TM mode, resulting in fractional bandwidths (FBW) of 50.56% and 59.31%, respectively.

The first simplification removed the solder mask layer entirely and additionally omitted any copper fill or ground-plane fill pattern that might surround the resonant element on the fabricated board but was not a part of the electromagnetically active split-ring geometry itself. It allowed to isolate the effect of a commonly made modelling shortcut: neglecting the solder mask and non-functional copper fill, which are sometimes omitted purely to reduce model complexity and mesh size, shortening simulation time.

**Figure 9:**
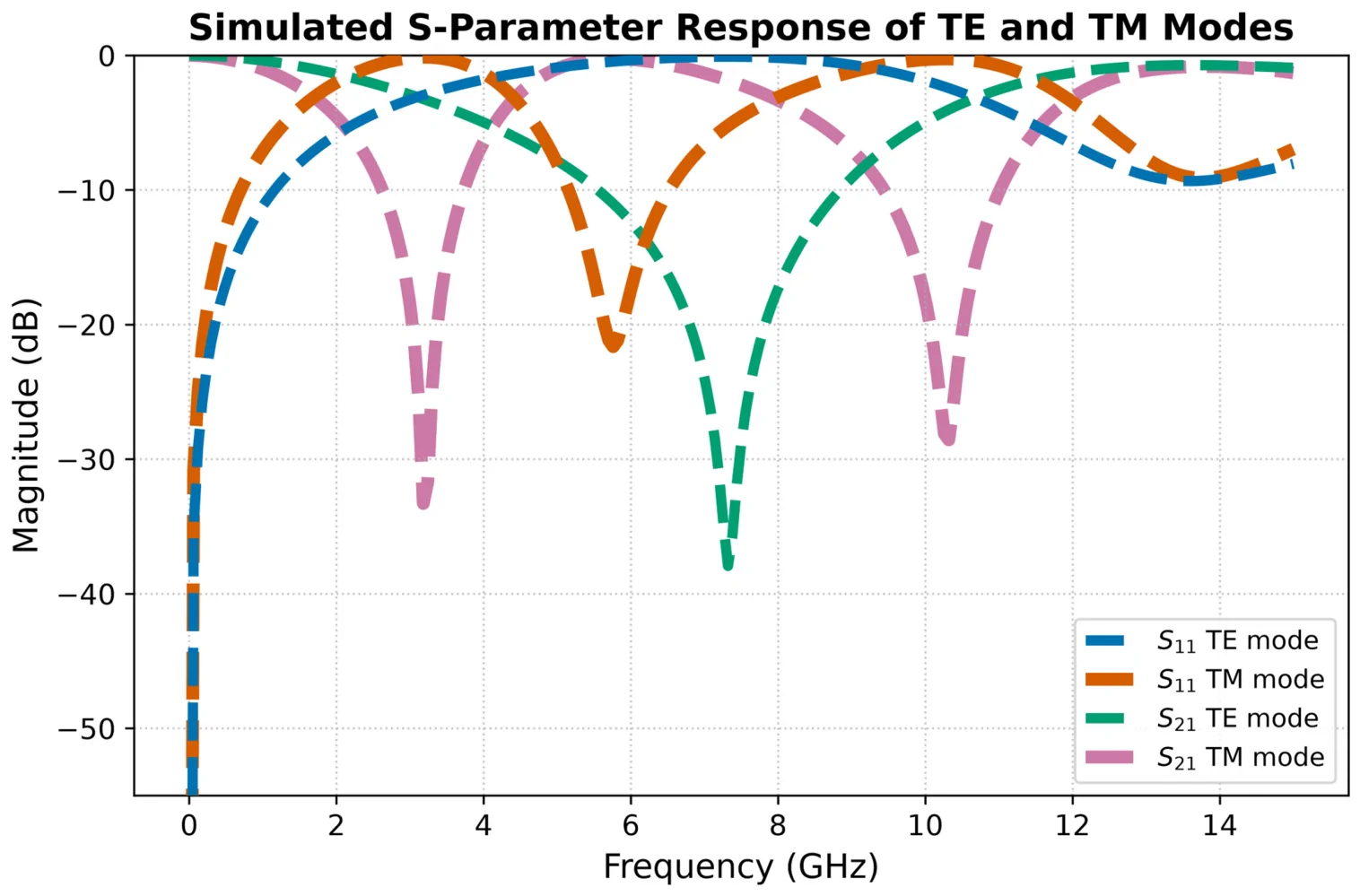
Simulated S11 and S21 response of the mask & fill free PCB model for TE and TM modes.

For the mask & fill free PCB model (Figure 9), resonance frequencies of 7.32 GHz (TE mode) and 3.18 GHz (TM mode) were observed in the S21 response, with corresponding attenuation depths of −37.94 dB and −33.29 dB, respectively. At the −10 dB level, the TE and TM modes exhibited bandwidths of 3.29 GHz and 1.11 GHz, respectively. These bandwidths corresponded to frequency ranges of 5.55–8.84 GHz for the TE mode and 2.63–3.73 GHz for the TM mode, resulting in fractional bandwidths (FBW) of 45.76% and 34.86%, respectively.

The second simplification (bare-copper model) removed the dielectric substrate from the model entirely, representing the split-ring conductor as a bare-copper trace suspended in the air. While physically unrealistic, this variant was included because it represented a modelling shortcut occasionally used when the dielectric properties of a substrate were not known with confidence.

**Figure 10:**
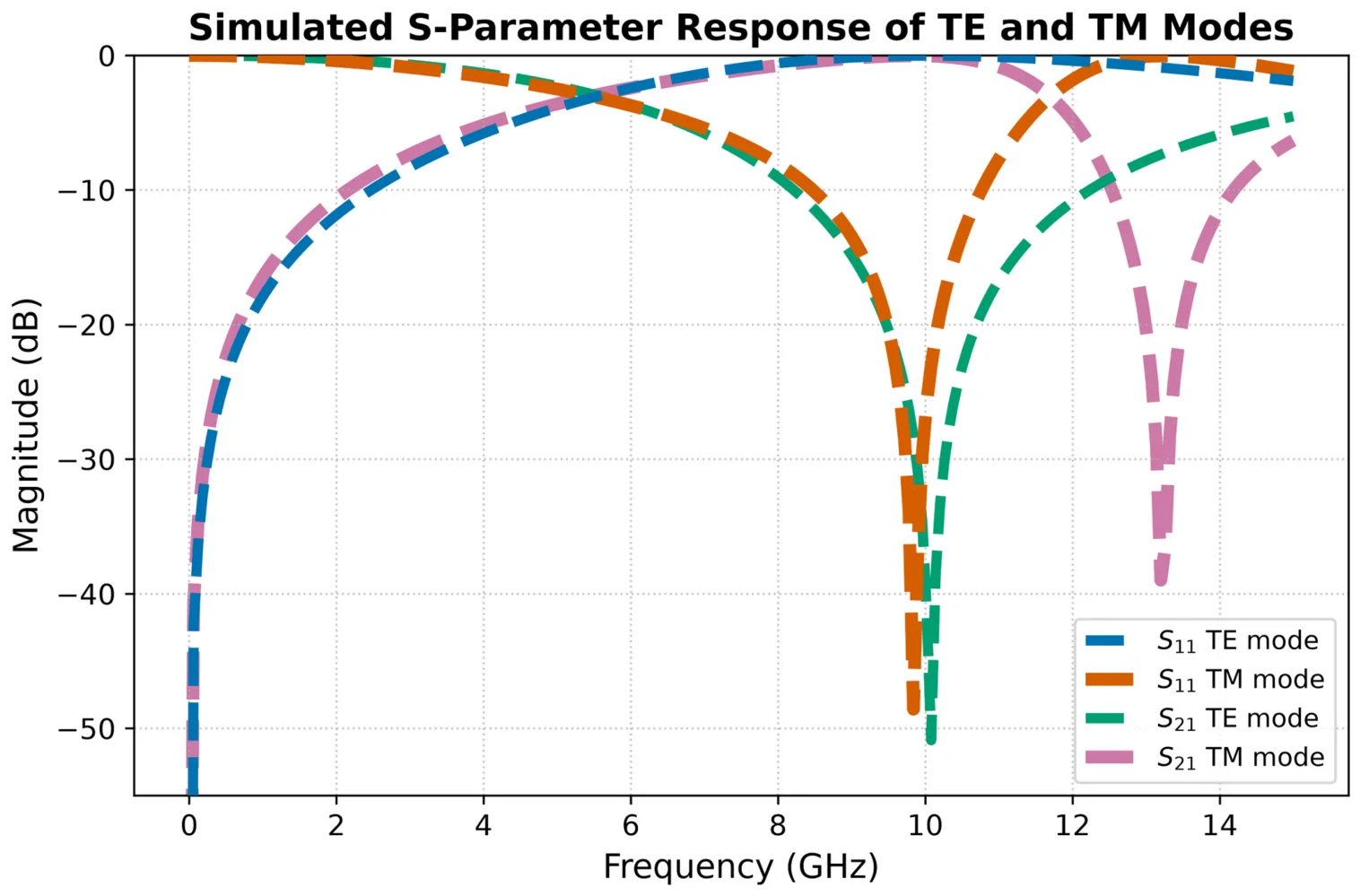
Simulated S11 and S21 response of the bare-copper PCB model for TE and TM modes.

Comparing this variant (Figure 10) with the full-stack structure (Figure 8), a direct quantitative comparison of the resonance parameters is not appropriate, as the two structures exhibit substantially different S21 characteristics. In particular, the resonances observed within the analysed frequency range in Figure 10 occur at frequencies that differ considerably from those observed in the full-stack model. This discrepancy may indicate that the observed resonances are associated with different electromagnetic responses rather than corresponding modes shifted in frequency. Therefore, the resonance frequencies, attenuation depths, and bandwidths should be interpreted in the context of this model individually, rather than directly compared as equivalent resonance characteristics.

For the bare-copper PCB model (Figure 10), resonance frequencies of 10.08 GHz (TE mode) and 13.20 GHz (TM mode) were observed in the S21 response, with corresponding attenuation depths of −50.88 dB and −38.99 dB, respectively. At the −10 dB level, the TE and TM modes exhibited bandwidths of 4.02 GHz and 1.66 GHz, respectively. These bandwidths corresponded to fractional bandwidths (FBW) of 39.36% for the TE mode and 12.39% for the TM mode. Unlike the full-stack and mask-and-fill-free PCB models, the bare-copper model exhibited a substantially different resonance pattern, indicating that removing the dielectric substrate changes the electromagnetic response and does not preserve the resonant behaviour observed in the other PCB configurations.

The comparison indicates that the dielectric substrate has a greater influence on the overall electromagnetic response than the omission of the solder mask and copper fill. When the dielectric substrate is retained, removing these secondary layers leaves the TE resonance frequency effectively unchanged, while the TM resonance shifts by 0.54 GHz and its attenuation depth changes by 3.88 dB. The TM bandwidth also decreases, indicating that the effect of these simplifications is polarisation dependent. Therefore, the omission of solder mask and copper fill may be acceptable for preliminary studies focused on the TE resonance frequency, but its suitability should be assessed separately when the TM response, resonance depth, or bandwidth is important.

### 3.2. Screen-Printing

Unlike the PCB case, where geometric and material details could be progressively removed from the baseline model, the screen-printing technology did not allow for an equivalent simplification approach. Instead of eliminating individual model features, the paste layer and textile substrate could be represented either as uneven surfaces or as homogeneous solid layers. Consequently, three model variants were developed for this technology, as summarised in Table 1.

The split-ring pattern was represented as a continuous paste layer of thickness 0.03 mm, printed on the textile with thickness equal to 1.6 mm.

**Figure 11:**
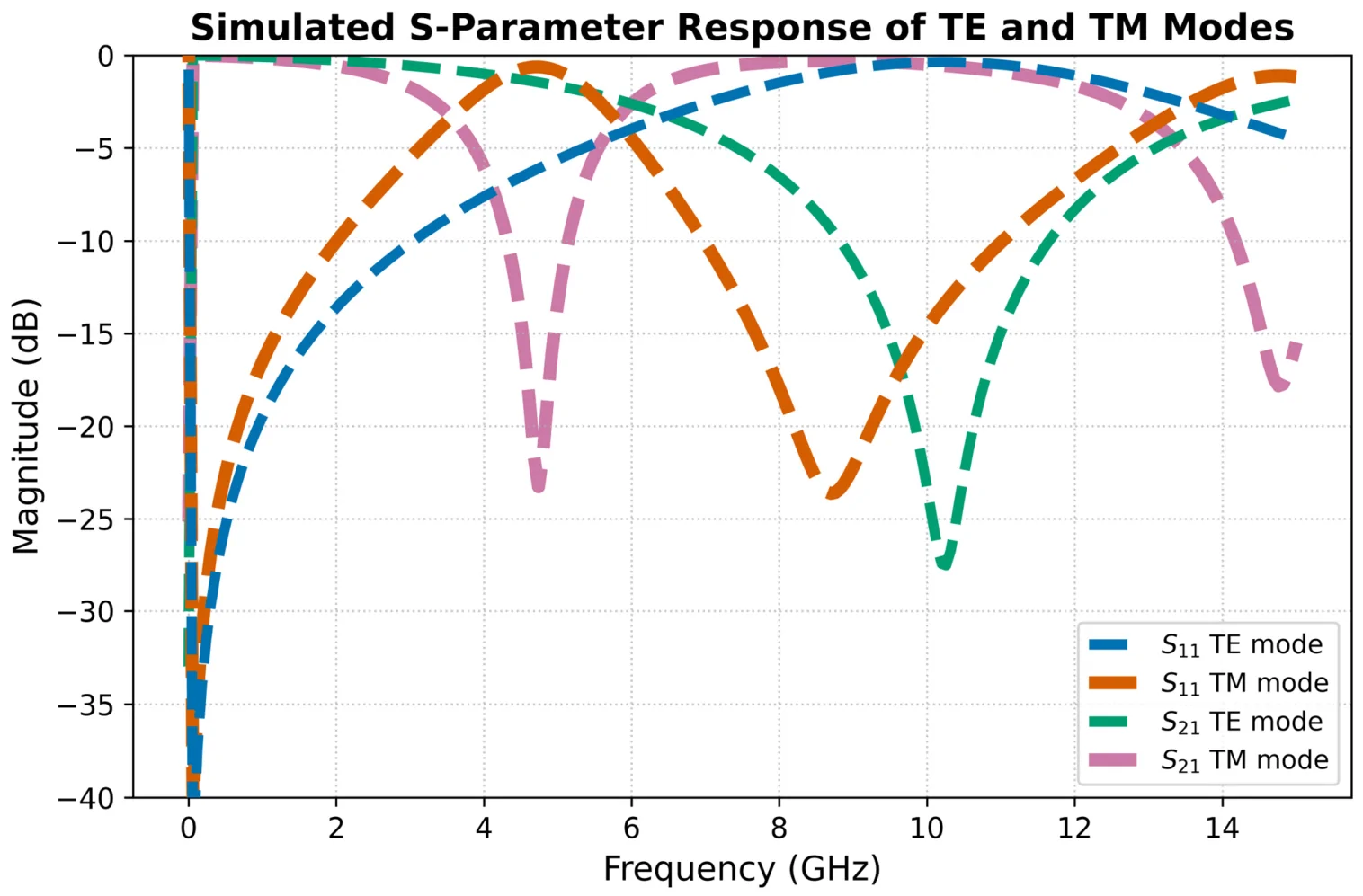
Simulated S11 and S21 response of the uneven surfaces, screen-printed model for TE and TM modes.

For the model with the most detail, resonance frequencies of 10.26 GHz (TE mode) and 4.74 GHz (TM mode) were observed in the S21 response presented in Figure 11, with corresponding attenuation depths of −27.50 dB and −23.26 dB, respectively. At the −10 dB level, the TE and TM modes exhibited bandwidths of 2.84 GHz and 0.82 GHz, respectively. These bandwidths corresponded to frequency ranges of 8.82–11.66 GHz for the TE mode and 4.32–5.14 GHz for the TM mode, resulting in fractional bandwidths (FBW) of 27.73% and 17.34%, respectively.

**Figure 12:**
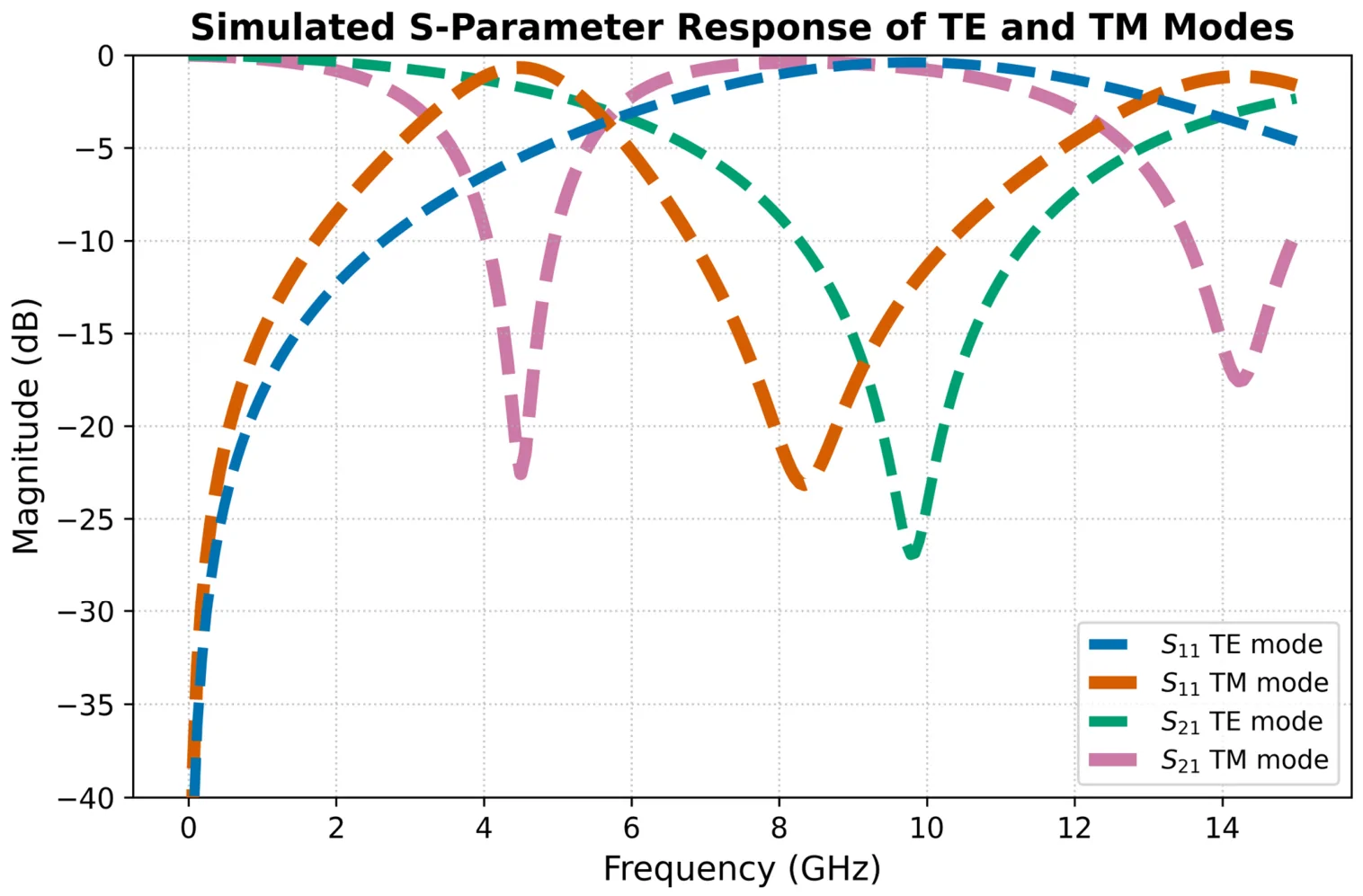
Simulated S11 and S21 response of the uneven paste-surface, screen-printed model for TE and TM modes.

For the uneven ink surface model (Figure 12), resonance frequencies of 9.78 GHz (TE mode) and 4.50 GHz (TM mode) were observed in the S21 response, with corresponding attenuation depths of −26.94 dB and −22.55 dB, respectively. At the −10 dB level, the TE and TM modes exhibited bandwidths of 3.07 GHz and 0.90 GHz, respectively. These bandwidths corresponded to frequency ranges of 8.28–11.35 GHz for the TE mode and 4.05–4.95 GHz for the TM mode, resulting in fractional bandwidths (FBW) of 31.28% and 20.0%, respectively.

**Figure 13:**
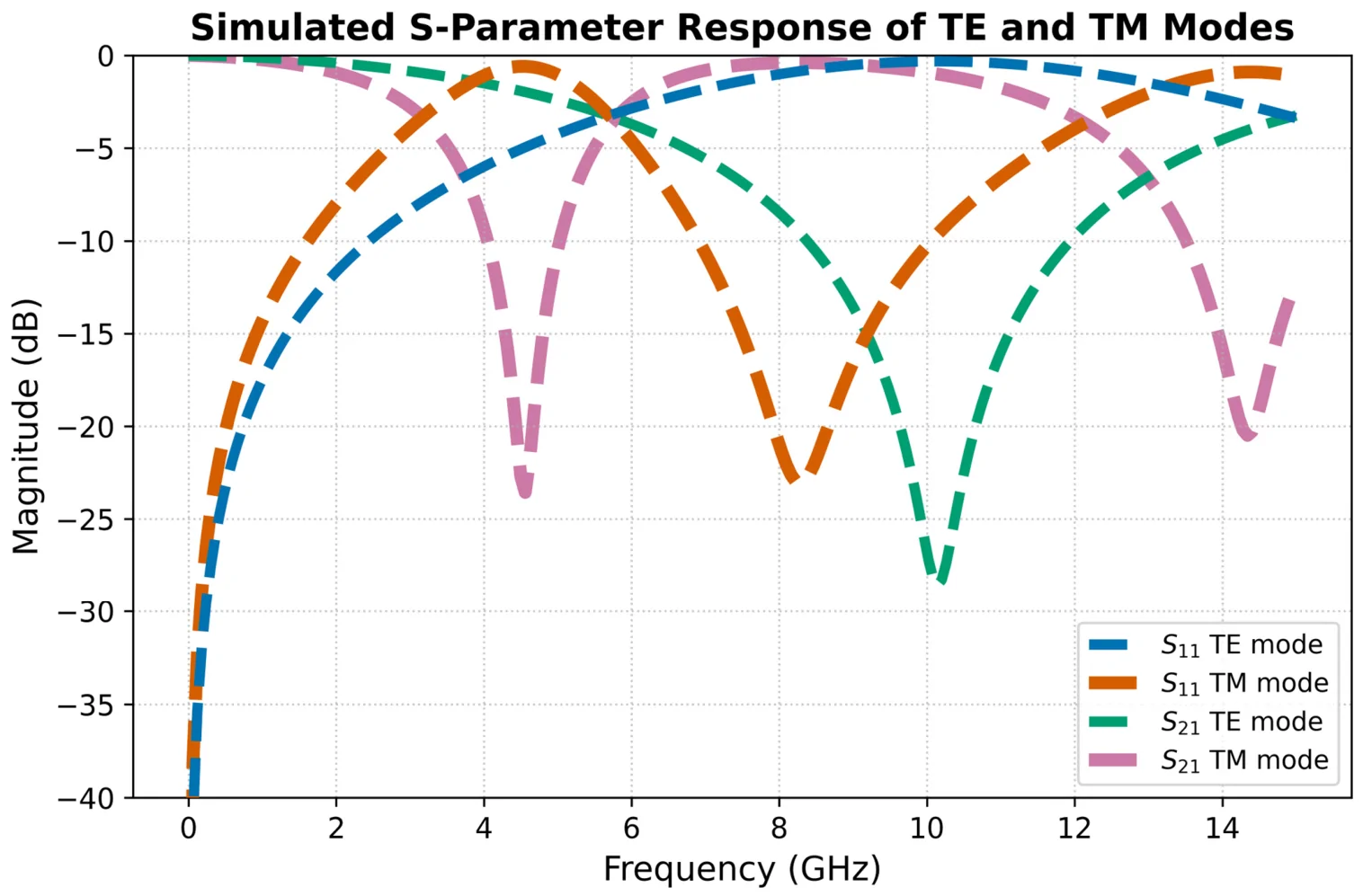
Simulated S11 and S21 response of the solid, screen-printed model for TE and TM modes.

For the solid, screen-printed model (Figure 13), resonance frequencies of 10.14 GHz (TE mode) and 4.57 GHz (TM mode) were observed in the S21 response, with corresponding attenuation depths of −28.57 dB and −23.34 dB, respectively. At the −10 dB level, the TE and TM modes exhibited bandwidths of 3.56 GHz and 0.98 GHz, respectively. These bandwidths corresponded to frequency ranges of 8.38–11.94 GHz for the TE mode and 4.05–5.03 GHz for the TM mode, resulting in fractional bandwidths (FBW) of 35.04% and 21.59%, respectively.

### 3.3. Embroidery

The embroidered realisation of the split-ring FSS represented the most challenging, in terms of geometric fidelity, fabrication route considered in this study. An embroidered conductor was intrinsically discontinuous at the level of the single stitch. The resonant pattern was normally realised as a sequence of conductive threads passing through the area of the material which create many cross-sections. In general, so many cross-sections could not be resolved within a periodic solver without incurring a prohibitive meshing and computational cost. Furthermore, the requirement in ANSYS HFSS for the geometry to be watertight made mesh generation and geometric integrity particularly challenging for structures with such a high level of geometric complexity. Therefore, the detailed-thread solid model was made by precise modelling of only electroconductive threads for the embroidery technology with regards to avoid creating intersections, which is problematic from the point of view of a numerical solver.

**Figure 14:**
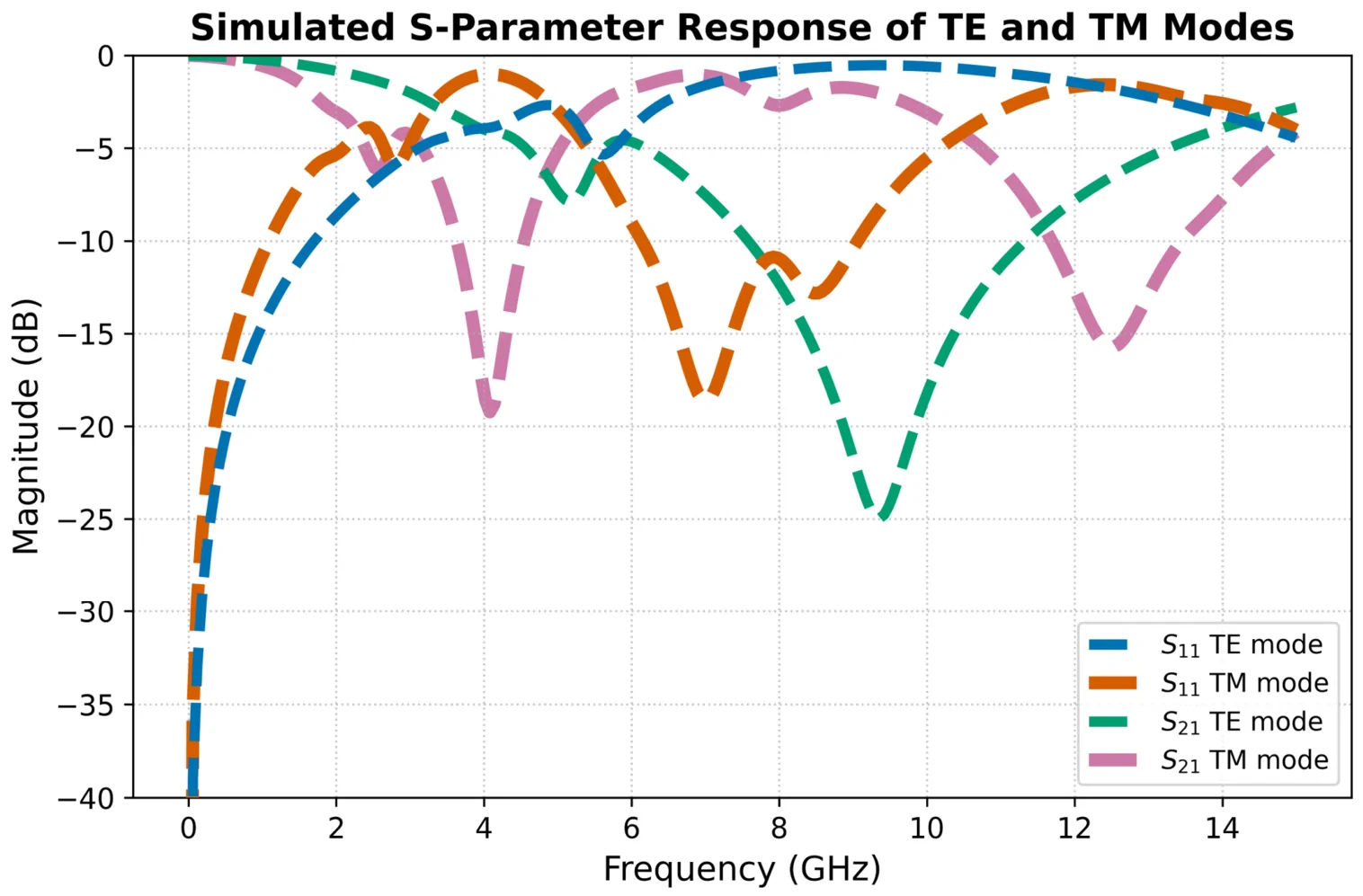
Simulated S11 and S21 response of the detailed-thread, embroidered model, for TE and TM modes.

For the most detailed model (Figure 14), resonance frequencies of 9.36 GHz (TE mode) and 4.08 & 12.48 GHz (TM mode) were observed in the S21 response, with corresponding attenuation depths of −24.96 dB and −19.22 & −15.72 dB, respectively. At the −10 dB level, the TE and TM modes exhibited bandwidths of 3.75 GHz and 0.92 & 1.76 GHz, respectively. These bandwidths corresponded to frequency ranges of 7.58–11.33 GHz for the TE mode and 3.63–4.55 & 11.69–13.45 GHz for the TM mode, resulting in fractional bandwidths (FBW) of 39.66% and 22.49 & 14.00%, respectively.

The more detailed embroidery pattern introduced additional electromagnetic coupling effects, resulting in more complex transmission characteristics compared with the less detailed structures. The additional resonances observed compared with the other models can be attributed to the closer spacing of the resonant features toward lower frequencies, causing subsequent resonances to become visible earlier within the analysed frequency range.

**Figure 15:**
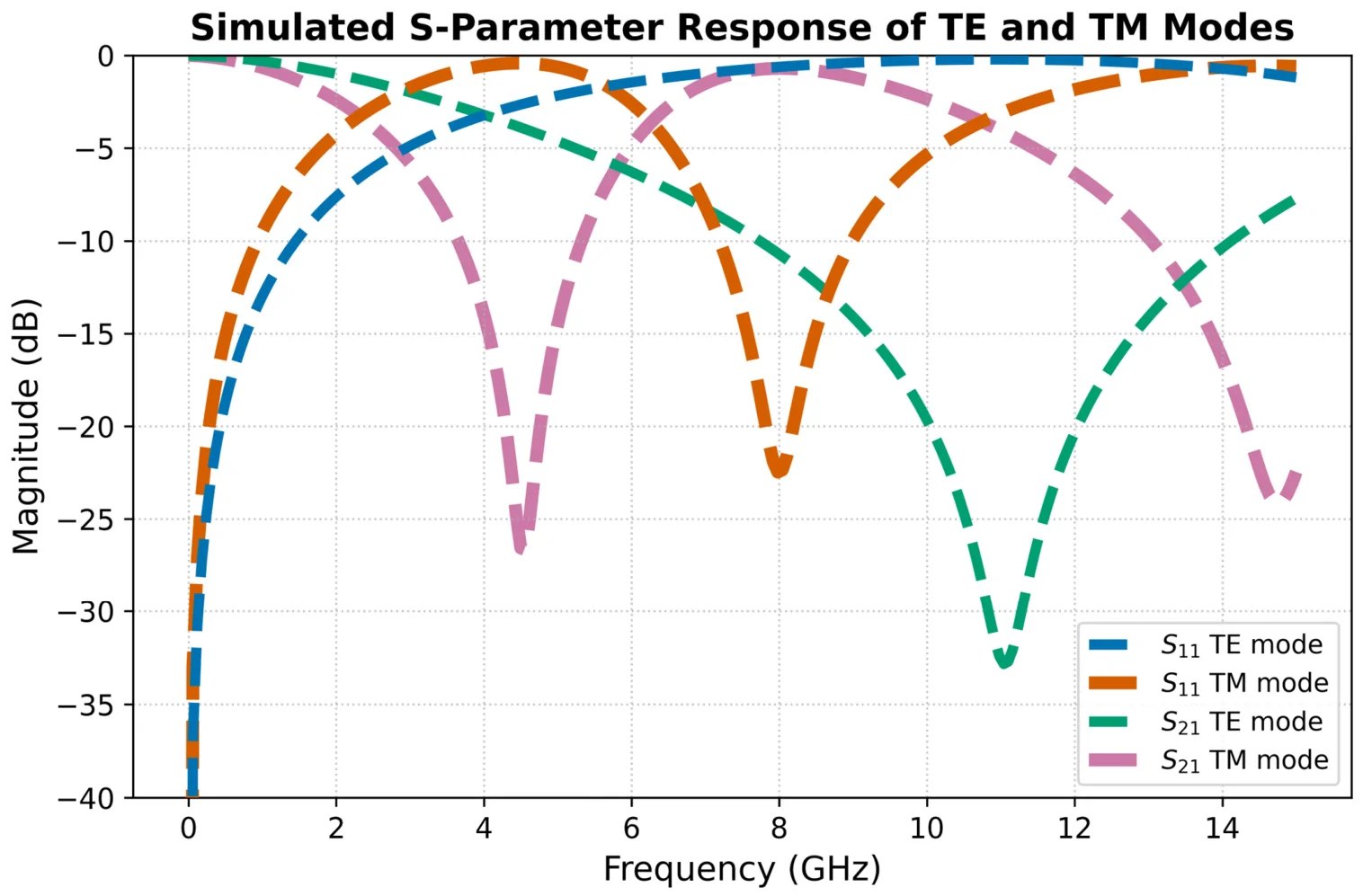
Simulated S11 and S21 response of the solid, embroidered model, for TE and TM modes.

For the solid, embroidered model, resonance frequencies of 11.04 GHz (TE mode) and 4.50 GHz (TM mode) were observed in the S21 response presented in Figure 15, with corresponding attenuation depths of −32.83 dB and −26.65 dB, respectively. At the −10 dB level, the TE and TM modes exhibited bandwidths of 6.38 GHz and 1.68 GHz, respectively, corresponding to frequency ranges of 7.74–14.12 GHz and 3.65–5.33 GHz. These values resulted in fractional bandwidths (FBW) of 58.37% for the TE mode and 37.42% for the TM mode, respectively.

Comparing this variant (Figure 15) with the reference case presented in Figure 14, a resonance frequency shift of Δf = 1.68 GHz for the TE mode and Δf = 0.42 GHz for the TM mode was observed, accompanied by response depth deviations of ΔdB = 7.87 dB and ΔdB = 7.43 dB, respectively. The resonance frequencies were shifted towards higher values, reaching 11.04 GHz for the TE mode and 4.50 GHz for the TM mode, while the attenuation depths increased to −32.83 dB and −26.65 dB, respectively.

The bandwidth of the resonant features was significantly broadened, increasing by approximately 70.1% for the TE mode and 82.6% for the TM mode relative to the reference case. The TE mode bandwidth expanded from 3.75 GHz (7.58–11.33 GHz) for the detailed embroidery model to 6.38 GHz (7.74–14.12 GHz) for the solid embroidery model, while the TM mode bandwidth increased from 0.92 GHz (3.63–4.55 GHz) to 1.68 GHz (3.65–5.33 GHz). These changes indicate that replacing the detailed embroidery pattern with a solid embroidered structure enhanced the electromagnetic interaction and resulted in stronger attenuation as well as broader resonance characteristics.

The final adaptive-pass results and associated convergence indicators are summarised in Table 3. The recorded values reflect the solver’s automatically controlled convergence process, with the reported termination status indicating that the applicable solver criteria were satisfied. The variation in final convergence indicators across the models is consistent with their differing geometric complexity and computational requirements.

**Figure 16:**
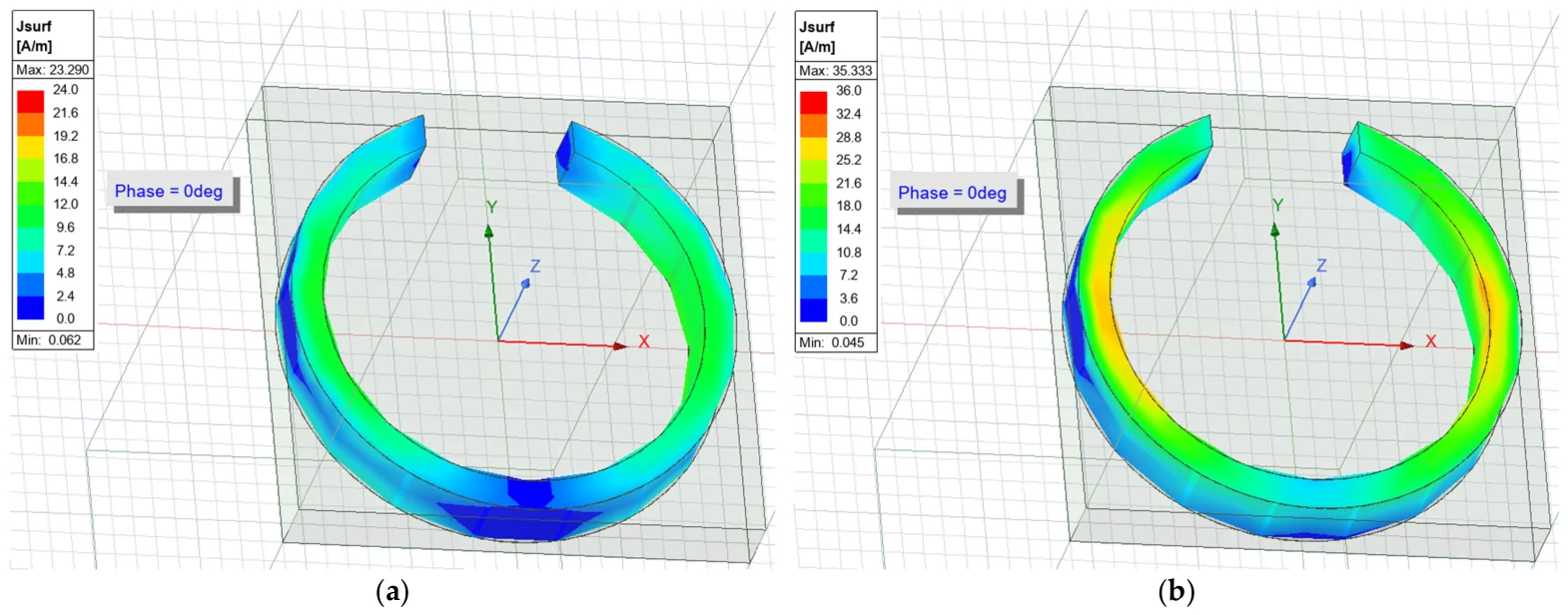
Surface-current density distributions of solid embroidery model for TM mode: 4.50 GHz (**a**) and TE mode: 11.04 GHz (**b**) at phase angle of 0°.

**Figure 17:**
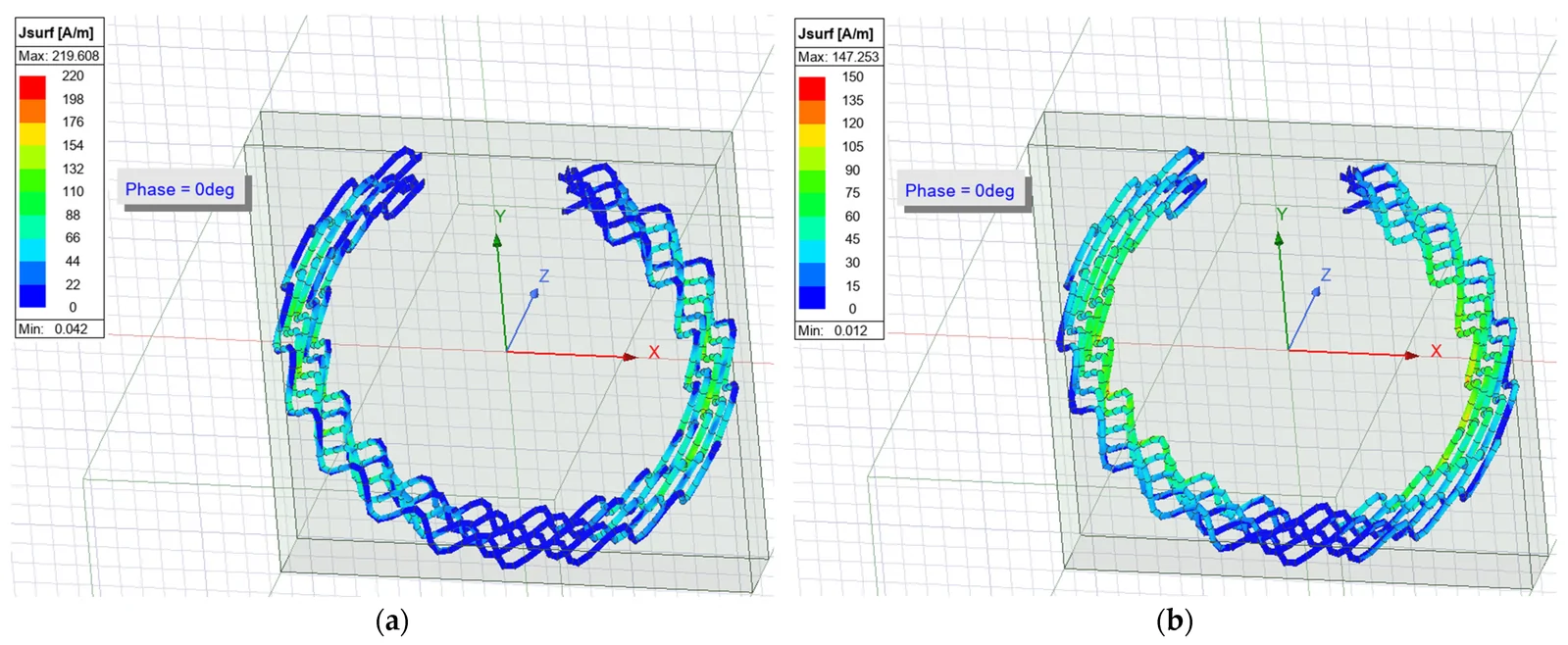
Surface-current density distributions of solid embroidery model for TM mode: 4.08 GHz (**a**) and TE mode: 9.36 GHz (**b**) at phase angle of 0°.

To provide a physical interpretation of the electromagnetic response observed in the S-parameter characteristics, the surface-current distributions were presented in Figure 16 for the solid embroidery model and Figure 17 for the detailed-thread embroidery model. While the S parameters described the reflection and transmission properties of the structure, the surface-current distributions revealed the resonant behaviour of the conductive elements and identify the regions where the electromagnetic energy was concentrated. The surface-current distributions were presented for corresponding resonance frequencies, for both modes at phase angle of 0°.

Surface-current distributions were evaluated using the same Floquet-port excitation of 1 W for all model variants. For comparison between models, the surface-current magnitude was normalised to the maximum value obtained within each corresponding model, allowing the spatial distribution of the induced currents to be compared independently of their absolute magnitude.

## 4. Discussion

The electromagnetic filtering behaviour of the FSS was evaluated from its simulated S parameters. The reflection, transmission, and absorption coefficients were obtained as $R=\;|S_{11}{|}^{2}$, $T=\;|S_{21}{|}^{2}$, and A=1−R−T, respectively [40]. These quantities were used to relate the simulated resonant responses to the functional behaviour of the FSS, i.e., whether incident electromagnetic energy was predominantly reflected, transmitted, or absorbed at a given frequency.

The present study was motivated by a practical modelling question: how sensitive the electromagnetic response is to geometric simplification. This question was answered by systematically reducing the fidelity of the same structure, allowing the influence of alternative modelling representations to be assessed. This approach provides a clearer understanding of which modelling assumptions have the greatest impact on simulated response and which may be acceptable during the early stages of design.

The results demonstrate that not all simplifications contribute equally to modelling error. Within the PCB models, removal of the solder mask produced minor changes, while omitting the dielectric substrate resulted in substantially larger deviations. This behaviour is consistent with electromagnetic theory, as the dielectric substrate directly influences the effective permittivity surrounding the resonator, thereby determining its electrical length and resonance conditions. Consequently, simplifications involving dielectric representation cannot be regarded as purely geometric approximations but instead modify one of the principal physical mechanisms governing FSS operation. This finding suggests that modelling strategies should prioritise detailed dielectric representation over secondary metallisation details when computational resources are limited.

An important observation was the different sensitivity of the TE and TM modes to identical modelling simplifications. A model that produced only minor deviations for one polarisation could already exhibit noticeable deviations for the other, indicating that modelling errors are inherently polarisation dependent. This justifies evaluating both modes independently rather than relying on a single aggregated error metric, which could conceal discrepancies that become important in practical applications. The observed TE/TM asymmetry is particularly notable because even the relatively simple PCB configuration, consisting of metallisation on only one side of the substrate, was sufficient to produce different modal behaviour. This suggests that asymmetry in material distribution alone may introduce polarisation-dependent effects that deserve greater consideration during model validation.

Among the analysed response parameters, bandwidth proved to be the most sensitive indicator of modelling fidelity. While resonance frequency and transmission attenuation generally changed in a gradual and predictable manner as detail was removed, bandwidth occasionally underwent qualitative changes. Such behaviour indicates that excessive simplification may alter the physical interpretation of the resonant response rather than merely reducing quantitative accuracy. Consequently, the bandwidth represents a particularly important parameter for evaluating whether a simplified model preserves the operating characteristics of the original structure. From the perspective of practical FSS manufacturing and application, maintaining the required operational bandwidth is generally more critical than preserving the exact magnitude of the transmission or reflection peak, as the bandwidth directly determines the frequency range over which the structure performs its intended function.

Comparison of the three fabrication technologies shows that the validity of model simplifications depends on the manufacturing method. Solid-conductor approximations commonly used for textile-based FSS may introduce different errors than those observed in PCB structures. The results also suggest that geometric adjustments may be needed to achieve comparable electromagnetic performance across screen-printing and embroidery, limiting the direct transfer of optimised geometries between technologies.

Despite their limitations, simplified models generally preserve the main trends in electromagnetic response. They therefore remain useful for preliminary design, optimisation, and parametric studies, where computational efficiency is a key consideration.

To facilitate a comprehensive comparison of the obtained results, all key parameters were consolidated. Table 4 and Table 5 summarise the simulation results and the differences between model variants.

The results demonstrate that the influence of model simplification depends strongly on the fabrication technology and on the type of electromagnetic response considered. The differences are particularly evident when comparing the detailed and solid embroidery models. For the embroidery structure, replacing the individual conductive threads with a solid conductor shifts the TE resonance frequency from 9.36 GHz to 11.04 GHz, corresponding to a shift of +1.68 GHz. The TM resonance at 4.08 GHz shifts to 4.50 GHz, corresponding to a shift of +0.42 GHz. The corresponding changes in S21 resonance depth are −7.87 dB for the TE mode and −7.43 dB for the TM mode, with the resonance depths changing from −24.96 dB to −32.83 dB and from −19.22 dB to −26.65 dB, respectively. These differences indicate that the thread-level geometry cannot be regarded solely as a geometrical refinement of the solid conductor.

A possible explanation is the non-uniform electromagnetic environment created by the individual conductive threads. In the detailed model, conductive and non-conductive regions are explicitly resolved, producing spatial variations in the effective electrical properties of the FSS. The resulting local electric and magnetic fields can interact with neighbouring thread segments, modifying the capacitive and inductive coupling within the resonant structure. In addition, the detailed representation defines the actual conductive topology and current paths, whereas the solid model assumes a continuous conductor. These differences can affect both the resonant frequency and the magnitude of the transmission response. The larger TE resonance frequency shift compared with the TM shift further indicates that the effect depends on the interaction between the thread arrangement and the incident field polarisation.

The detailed embroidery model also exhibits an additional TM resonance at 12.48 GHz, with an attenuation depth of −15.72 dB, which is not present in the solid representation. This resonance may originate from localised current distributions and near-field coupling associated with the thread geometry. Since only S-parameter responses are considered here, these mechanisms cannot be identified unambiguously without corresponding electric-field, magnetic-field, or surface-current distributions.

The screen-printed structures show a different sensitivity pattern. Comparing the uneven paste-surface model with the solid representation, the TE resonance frequency shifts from 9.78 GHz to 10.14 GHz, corresponding to a change of 0.36 GHz, while the TM resonance shifts from 4.50 GHz to 4.57 GHz, corresponding to a change of 0.07 GHz. The corresponding changes in resonance depth are 1.63 dB for TE and 0.79 dB for TM, with the attenuation depths changing from −26.94 dB to −28.57 dB and from −22.55 dB to −23.34 dB, respectively. This suggests that the representation of the conductive layer and its surface geometry can influence the resonance frequency and attenuation depth, although the magnitude of these effects differs between the two modes. In contrast to embroidery, the screen-printed conductive region remains a continuous layer, so the simplification does not alter the basic conductive topology to the same extent.

For the PCB models, the comparison indicates that the dielectric substrate has a substantially greater influence on the electromagnetic response than the omission of the solder mask and copper fill. Removing these secondary layers while retaining the dielectric substrate leaves the TE resonance frequency unchanged and shifts the TM resonance frequency by 0.54 GHz. The corresponding changes in resonance depth are 0.08 dB for the TE mode and 3.88 dB for the TM mode. In contrast, removing the dielectric substrate produces a substantially different resonance pattern, with resonances that cannot be unambiguously matched to the corresponding resonances of the full-stack PCB model. Therefore, the bare-copper results should be interpreted as a different electromagnetic response rather than as a direct frequency shift of the reference resonances. The results indicate that the dielectric substrate has a substantial influence on the resonant behaviour of the investigated PCB structure. Although omitting the solder mask and copper fill has a limited effect on the TE response, the observed changes in TM resonance frequency, attenuation depth, and bandwidth indicate that these layers may still affect the simulated response. Therefore, their omission in preliminary simulations should be considered in relation to the required modelling accuracy and the electromagnetic characteristics of interest.

These observations have direct implications for the modelling workflow. A single level of model fidelity should not be assumed to be appropriate for all fabrication technologies. For PCB structures, a simplified model retaining the dielectric substrate can be employed during initial design studies and parametric optimisation, while the full layered model can be adopted for higher-fidelity comparative numerical investigations of fabrication-related effects. For screen-printing, a solid conductive representation is suitable for initial design studies, whereas the uneven paste-surface model enables assessment of the simulated electromagnetic effects associated with surface irregularities introduced during fabrication. For embroidery, the solid representation can be used for initial dimensional exploration, while the detailed thread-level model enables investigation of the effects of thread spacing, crossings, and conductive-path topology on the simulated electromagnetic response. These observations are specific to the investigated structure and should be interpreted as comparative numerical observations within the adopted modelling framework rather than experimentally validated design guidelines.

## 5. Conclusions

This study examines how the level of geometric and material fidelity in modelling a split-ring FSS influences the simulated S11 and S21 responses across three fabrication technologies (PCB, screen-printing, and embroidery) for a common unit-cell geometry. The models were deliberately simplified in stages to investigate which fabrication-related features can be omitted at different stages of the design process without substantially changing the simulated electromagnetic response.

The results suggest that the impact of modelling simplifications depends strongly on both the fabrication technology and the scope of the numerical investigation. Consequently, different levels of geometric representation may be appropriate for different stages of comparative numerical studies. The observed sensitivity to model simplification varies substantially among the investigated technologies, indicating that fabrication-related geometric features do not affect all implementations to the same extent.

Within the PCB technology, the dielectric substrate is found to be the dominant modelling factor. In contrast, omission of the solder mask and copper fill produces only a minor change in the investigated bandwidth. This indicates that these secondary layers can be omitted during initial design and parametric optimisation while retaining the dielectric substrate, allowing a simpler and faster model to be used without substantially altering the simulated operating band. The full layered representation may nevertheless be employed when a more detailed fabrication-aware numerical representation is desired. The results suggest that, for the investigated PCB FSS structure, the simulated electromagnetic response is more strongly influenced by the dielectric configuration than by the inclusion of secondary conductive layers.

The textile technologies require a different modelling approach. For screen-printing, replacing the uneven conductive and textile surfaces with solid representations results in an average absolute bandwidth difference of 0.44 GHz at the level of −10 dB. This indicates that a solid representation can provide a useful compromise between computational efficiency and electromagnetic fidelity during initial design, while surface-aware modelling becomes more appropriate as the design is refined and when fabrication-related geometric effects are investigated. The relatively limited bandwidth change also suggests that the detailed surface representation does not necessarily need to be introduced at the earliest stage of the design process.

For embroidery, the discrete conductive thread structure has a substantially greater influence on the simulated response. Replacing the detailed-thread geometry with a solid conductor results in an average absolute bandwidth difference of 1.7 GHz at the −10 dB level. Consequently, although the solid representation remains useful for rapid initial dimensional exploration, it should not be relied upon for establishing final resonant dimensions. Detailed thread-level modelling may be beneficial during more advanced numerical investigations when the influence of embroidery-specific geometric features is of interest. The additional resonances observed in the detailed embroidery model, which are absent from the solid representation, indicate that geometric simplifications may alter the simulated electromagnetic response beyond a simple shift of the dominant resonance. These additional resonances may be associated with specific structural features of the embroidery, such as electrical path length variations, localised capacitive coupling, or contact conditions between yarn. The physical origin of these additional resonances requires further investigation.

The comparison of the three fabrication technologies indicates that a modelling simplification that produces only minor changes in the simulated response of a rigid PCB structure may lead to substantially larger variations in textile FSS models. The observed bandwidth differences increase from PCB through screen-printing to embroidery, indicating progressively greater sensitivity to the geometric representation of the conductive structure within the adopted numerical framework. Consequently, the appropriate level of model complexity depends on the electromagnetic influence of the fabrication-related features being represented.

The comparison between screen-printed and embroidered implementations also indicates that the same nominal FSS geometry may not produce an identical electromagnetic response when realised using different fabrication technologies. Slight technology-specific modification of the resonator dimensions may therefore be required to obtain a comparable target response. The fabrication technology should consequently be considered already during electromagnetic optimisation rather than treated only as a manufacturing step following completion of the design.

Several limitations should be considered when interpreting these findings. First, all conclusions are based exclusively on numerical simulations, and no fabricated PCB, screen-printed, or embroidered prototypes were experimentally tested. Consequently, the study establishes the internal consistency of different modelling fidelities relative to the most detailed numerical models rather than their absolute agreement with physical measurements. Second, all simulations assumed normal incidence, leaving the influence of oblique incidence and polarisation-dependent angular effects for future investigation. These limitations are particularly relevant for textile-based FSS, where mechanical flexibility and structural deformation may further influence electromagnetic performance.

In addition, the detailed embroidery model should be interpreted with caution. The thread density and intersections were constrained by the requirements of the electromagnetic modelling software, and it therefore cannot be established with complete certainty that the detailed representation corresponds to a sufficiently dense embroidered structure for direct comparison with the simplified solid model. Experimental verification is required to resolve this uncertainty.

The bandwidth differences reported in this study should be regarded as case-specific reference values for the investigated split-ring geometry, materials, and modelling conditions rather than universal accuracy thresholds. A different resonator geometry, substrate, operating frequency, or fabrication process may exhibit different sensitivity to model simplification. Nevertheless, the results suggest that different levels of model complexity may be appropriate for different numerical investigations. While simplified representations offer reduced computational cost, more detailed fabrication-aware models can reveal electromagnetic features that may not be captured by simplified geometric descriptions.

Future work should prioritise experimental fabrication and measurement of representative PCB, screen-printed, and embroidered samples to validate the simulated data against measured responses. Such validation would establish whether the observed modelling deviations remain representative under practical fabrication conditions and could provide a basis for defining experimentally supported modelling tolerances. Further investigation should also address oblique incidence, polarisation-dependent angular effects, and mechanically deformed textile structures to determine the applicability of the proposed modelling observations reported in this study.

## Figures and Tables

**Table 1:** Table presenting design of experiment.

Technology	Model Variant Name	Substrate Representation ^1^	Conductor Representation ^2^
PCB	full stack ^3^	dielectric with solder mask	solid
	mask & fill free	dielectric, no mask & fill	solid
	bare copper	none	solid
Screen-printing	uneven surfaces	uneven	uneven
	uneven paste surface	solid	uneven
	solid ^4^	solid	solid
Embroidery	detailed thread	solid	detailed thread
	solid	solid	solid

^1^ Description of non-conductive material representation. ^2^ Geometric representation of the conductive material. ^3^ Layers of the PCB board stacked on top of each other. ^4^ Solid refers to approximating the complicated shape with cuboid.

**Table 2:** Material parameters.

Material	Relative Permittivity	Dielectric Loss Tangent	Relative Permeability	Bulk Conductivity
Copper	1	0	0.999991	58,000,000 S/m
FR4 (dielectric)	4.4	0.02	1	0
Core (dielectric)	4.5	0.02	1	0
Cotton	1.9	0.055	1	0
Silver threads	1	0	1	100,000 S/m
Silver paste	1	0	1	1,000,000 S/m

**Table 3:** Numerical convergence statistics for the HFSS simulation.

Technology View	Model Variant Name	Total Passes	Final Solved Elements	Final Max Mag. ∆S	Status
PCB 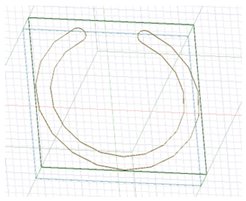	full stack	6	6855	0.06	converged
	mask & fill free	4	4067	0.08	converged
	bare copper	5	4220	0.06	converged
Screen-printing 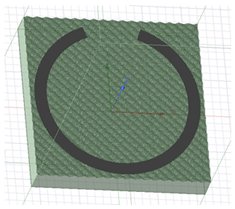	uneven surfaces	3	330,160	0.02	converged
	uneven paste surface	3	97,482	0.05	converged
	solid	3	2481	0.05	converged
Embroidery 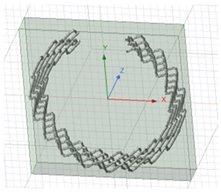	detailed thread	3	325,679	0.003	converged
	solid	3	2757	0.08	converged

**Table 4:** Summary table.

Technology View	Model Variant Name	Bandwidth ^1^ [GHz]	FBW ^1^ [%]	Simulation Time ^2^	TE S21 Peak [GHz, dB]	TM S21 Peak [GHz, dB]
PCB 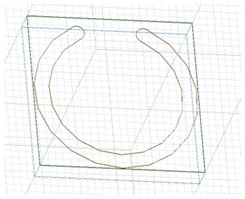	full stack	3.62/1.58	50.56/59.31	1 min 35 s	7.32, −37.86	2.64, −37.17
	mask & fill free	3.29/1.11	45.76/34.86	50 s	7.32, −37.94	3.18, −33.29
	bare copper ^3^	4.02/1.66	39.36/12.39	55 s	10.08, −50.88	13.20, −38.99
Screen-printing 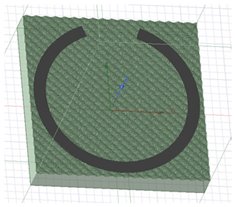	uneven surfaces	2.84/0.82	27.73/17.34	19 min 53 s	10.26, −27.50	4.74, −23.26
	uneven paste surface	3.07/0.90	31.28/20.00	4 min 57 s	9.78, −26.94	4.50, −22.55
	solid	3.56/0.98	35.04/21.59	37 s	10.14, −28.57	4.57, −23.34
Embroidery 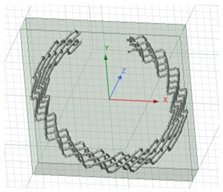	detailed thread ^4^	3.75/0.92	39.66/22.49	~10 h	9.36, −24.96	4.08, −19.22
	solid	6.38/1.68	58.37/37.42	32 s	11.04, −32.83	4.50, −26.65

^1^ The bandwidth and FBW values are reported for the TE and TM modes, respectively, following the indicated order. ^2^ The reported simulation times are approximate and serve only to indicate the relative computational effort, as they are not fully repeatable. The simulation time also depends on the solver settings. ^3^ The bare-copper PCB model is excluded from direct comparison because its S21 response does not exhibit corresponding resonances within the analysed frequency range, making the comparison of resonance-related parameters inapplicable. ^4^ Embroidery results demonstrate the differences between two alternative embroidery modelling approaches rather than the error of a simplified model relative to a validated physical reference. Consequently, the findings indicate that continuous solid-conductor representation may not reproduce all electromagnetic features observed in the detailed thread-level model. Additional resonance peak for detailed-thread model was not included in the table bandwidth comparison, as direct correlation to the resonance peaks of other simulation models presented in the table is unlikely.

**Table 5:** Comparison table.

Model Variant Name	∆Bandwidth [GHz]	∆FBW [%]	∆TE Frequency [GHz]	∆TE Peak [dB]	∆TM Frequency [GHz]	∆TM Peak [dB]
full stack	0.00/0.00	0.00/0.00	0.00	0.00	0.00	0.00
mask & fill free	−0.33/−0.47	−4.80/−24.45	0.00	−0.08	+0.54	+3.88
bare copper	N/A ^1^	N/A	N/A	N/A	N/A	N/A
uneven surfaces	0.00/0.00	0.00/0.00	0.00	0.00	0.00	0.00
uneven paste surface	+0.23/+0.08	+3.55/+2.66	−0.48	+0.56	−0.24	+0.71
solid	+0.72/+0.16	+7.31/+4.25	−0.12	−1.07	−0.17	−0.08
detailed thread	0.00/0.00	0.00/0.00	0.00	0.00	0.00	0.00
solid	+2.63/+0.76	+18.71/+14.93	+1.68	−7.87	+0.42	−7.43

^1^ Not applicable.

## Data Availability

The raw data supporting the conclusions of this article will be made available by the authors on request.
